# Development and Advantages of Biodegradable PHA Polymers Based on Electrospun PHBV Fibers for Tissue Engineering and Other Biomedical Applications

**DOI:** 10.1021/acsbiomaterials.1c00757

**Published:** 2021-10-15

**Authors:** Łukasz Kaniuk, Urszula Stachewicz

**Affiliations:** AGH University of Science and Technology, Faculty of Metals Engineering and Industrial Computer Science, al. A. Mickiewicza 30, 30-059 Kraków, Poland

**Keywords:** biodegradable, polymers, PHA, electrospinning, PHBV

## Abstract

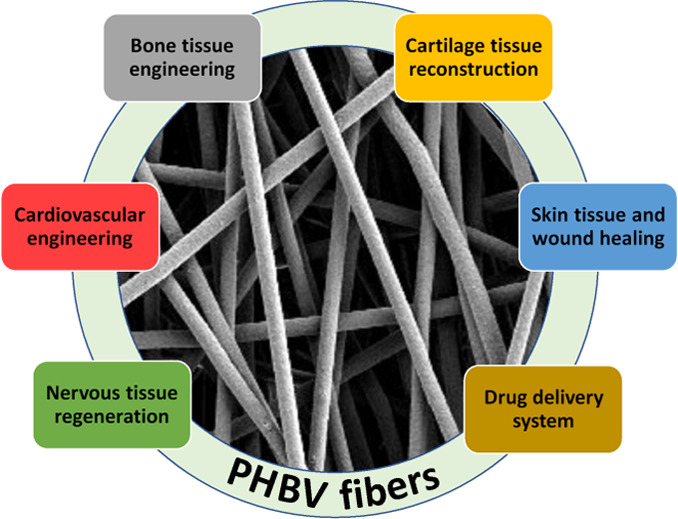

Biodegradable polymeric biomaterials offer a significant advantage in disposable or fast-consuming products in medical applications. Poly(3-hydroxybutyrate-*co*-3-hydroxyvalerate) (PHBV) is an example of a polyhydroxyalkanoate (PHA), i.e., one group of natural polyesters that are byproducts of reactions taking place in microorganisms in conditions with an excess carbon source. PHA polymers are a promising material for the production of everyday materials and biomedical applications. Due to the high number of monomers in the group, PHAs permit modifications enabling the production of copolymers of different compositions and with different proportions of individual monomers. In order to change and improve the properties of polymer fibers, PHAs are combined with either other natural and synthetic polymers or additives of inorganic phases. Importantly, electrospun PHBV fibers and mats showed an enormous potential in both the medical field (tissue engineering scaffolds, plasters, wound healing, drug delivery systems) and industrial applications (filter systems, food packaging). This Review summarizes the current state of the art in processing PHBV, especially by electrospinning, its degradation processes, and biocompatibility studies, starting from a general introduction to the PHA group of polymers.

## 1 Introduction

Polymeric biodegradable biomaterials offer a significant advantage in medical applications thanks to their ability to break down and be removed after serving their purpose. Their medical applications include surgical sutures, implants, drug delivery, and regenerative medicine. The degradability and recyclability of protective clothing, especially in a pandemic situation such as the current one with COVID-19,^1,2^ are of great importance for medical waste management. The global problem of waste disposal has contributed to the search for solutions using biodegradable polymers with properties comparable to petroleum materials. For several decades, the biodegradation potential has become an important factor for matrices, especially for disposable or fast-consuming products. Solutions have also been sought in materials of natural origin, but often, their mechanical properties have not been satisfactory for either producers or users. The culminating point in plastics processing was the exploration of polylactide, a fully biodegradable polymer made from cornmeal,^3,4^ which was obtained by DuPont in the first half of the 19th century. This discovery resulted in a growing interest in biodegradable polymers and the desire to patent new forms of these materials. Currently, the most well-known and frequently used polymers in many fields are the already-mentioned polylactide (PLA), polyglycolide (PGA), polycaprolactone (PCL), and the whole group of PHAs. The latter group includes the widely used polyhydroxybutyrate (PHB). This is a biodegradable thermoplastic polyester, produced and stored by various species of bacteria, which has been introduced onto the plastics market.^4−9^ In the case of medical devices, the properties of the materials used are extremely important, especially in the case of degradable materials, as the degradation time of an implant, for instance, should be sufficient for the material to support tissue regeneration.^10−13^ The advantage of PHAs comes from (i) the possibility to produce them via enzymatic synthesis employing microorganisms and (ii) their complete biodegradability compared to other degradable polymers.^14^ Polymers frequently used in medicine and tissue engineering are the previously mentioned PLA,^15^ PCL,^16^ PGA,^17^ and a copolymer of PGA and PLA (poly(lactic-*co*-glycolic acid), PLGA).^18^ PLA is produced by lactic acid fermentation and polymerization and is completely degradable. In addition, it has mechanical properties comparable to PHB, whose parameters depend on the molecular weight of the polymers.^11,14,19−21^ PCL, as a semicrystalline, synthetic polyester, is considered to be a nontoxic and tissue-compatible polymer; however, it degrades more slowly than PLA.^11,14,19^ On the other hand, PGA is a highly crystalline, biodegradable polyester.^22^ Polymer crystallinity significantly affects the degradation of the material; therefore, with a view to reduce the degree of crystallinity, PLGA was tested.^11,14,19^ The degradation of biodegradable polymers, the methods of production, and the processing of these materials are extremely important factors to determine ecological efficiency. PLA and PCL are frequently used biodegradable polymers, even though they are prone to hydrolytic degradation during processing.^23^ Reports from literature^23^ confirm that the enzymatic degradation of biodegradable polymers is more desirable due to the rapid degradation time of the material, while a hydrolytic degradation may take up to several years. Moreover, much research has been focused on naturally-based materials such as collagen,^24−26^ keratin,^27−29^ and other proteins,^30,31^ as well as alginate,^32−34^ cellulose,^35−38^ and other polysaccharides,^30,31^ whereas the group of natural polymers described in this Review are PHAs.

Polymers from the PHA group can be produced by many types of bacteria using (i) external renewable energy sources: glucose, sucrose, triglycerides, starches, or cellulose; (ii) production waste: whey, molasses, glycerol, or cereal bran; (iii) fossil resources: mineral oil, methane, or coal; (iv) sewage and municipal waste; (v) organic acids: hydroxybutyric acid or propionic acid.^3,10,39−41^ The production process and biodegradation properties of these polymers led to an increase in their use. The properties of PHB polymers made it possible to process them using many different methods, injection, foil blowing, or thermoforming, while their production costs, relatively low efficiency of processes, and high brittleness of the material proved inconvenient. The desire to improve PHB properties and broaden their applications led to the introduction of material changes.^3,6−9,12,21^ An approach that has been known for a long time is the creation of composites, i.e., reinforced materials, whose matrix may be a polymer. Another way to change polymer properties is copolymerization,^8,21^ thus making it possible to produce materials with an appropriate proportion of monomers and modify their physicochemical properties. There are many copolymers in the PHA group that are made of chemically linked hydroxybutyrate (HB), hydroxyvalerate (HV), or hydroxyhexanoate (HHx) monomers.^21,42^ This Review focuses on a comparison of their properties and applications, especially in PHBV. PHBV copolymer has many advantages over other types of PHA polymers such as toughness and elasticity.^43^ To obtain the desired properties of polymeric materials, surface or volume modifications, e.g., copolymers or blends with various organic and inorganic additives, can be introduced. The variety and wide range of plastic modifications make their subsequent applications easier.^6,9,21,44^ Products based on PHAs are used for the production of packaging as well as medical materials. Potential biomedical applications include: implants of various tissues, sutures, patches, stents, and matrices for the drug release system. PHA-based materials have a great potential in terms of biocompatibility and biodegradation.^4,6,12,42,44,45^

PHA polymers are a promising material for the production of everyday materials and biomedical applications. The variety of polymers in this group and the possibility of using their combinations as copolymers are very important factors. This Review begins with general information on the PHA polymer group. The characteristics and physicochemical parameters of individual PHA polymers are determined. The degradation processes and biocompatibility of polymers are described in detail. However, this Review focuses on PHA materials and provides detailed information summarizing all PHBV treatments, especially with electrospun fibers, with a summary of all the parameters used for electrospinning. All the parameters of the PHBV fibers reported on thus far were analyzed, showing their high dispersion in the current production of solid fibers. Importantly, we indicated all biocompatibility studies, dividing them into *in vitro* studies using particular cell lines and *in vivo* studies using specific animal models. The advantages and variety of electrospun meshes have made possible the wide application of nonwovens in many industries. Products made of PHA, in particular PHBV fibers, are most widely used in biomedical applications.

## 2 Polyhydroxyalkanoates: Types of Bacterial Polymers

**Figure 1:**
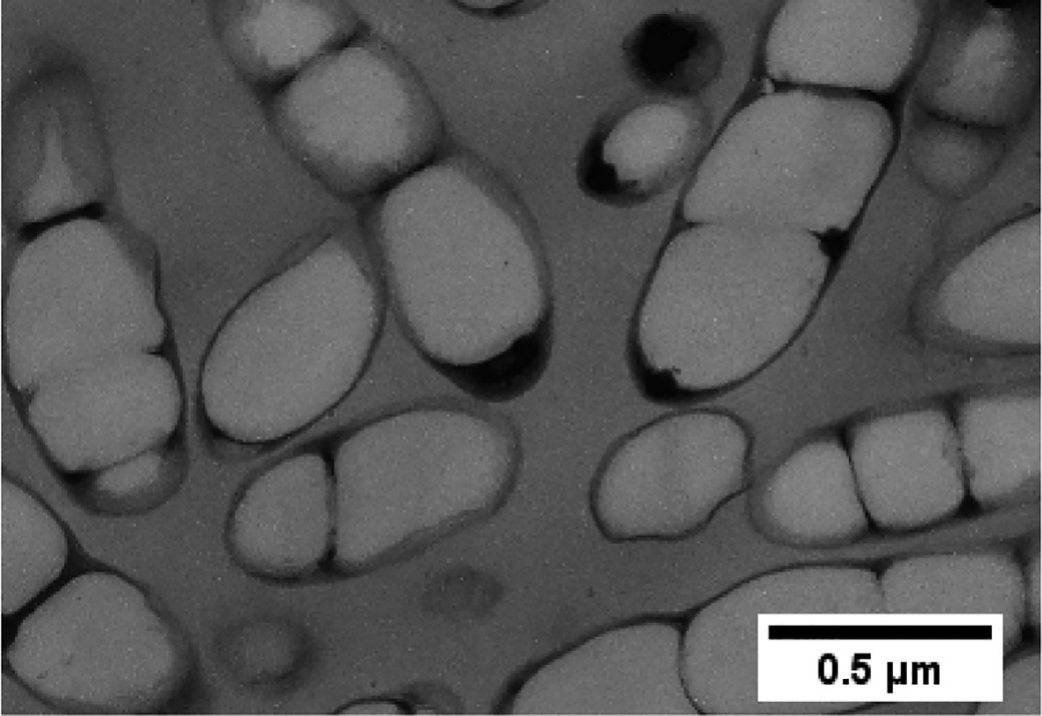
Transmission electron micrograph of a thin section of recombinant *Ralstonia eutropha* containing amounts of P(3HB-*co*-3HHx). Reproduced with permission from ref (52). Copyright 2000 Elsevier.

PHAs are a group of natural polyesters mainly made of carbon, hydrogen, and oxygen atoms. They are synthesized by microorganisms as a result of the fermentation of at least 75 different species of bacteria.^4,46−49^ PHA is produced intracellularly in the presence of excess carbon in a situation of limited nutrients (oxygen, phosphorus, and nitrogen) (Figure 1).^4,6,48,50−53^ They are produced in the form of solid, undissolved cytoplasmic inclusions and can accumulate at high concentrations because they do not affect the osmosis.^6,50,52−55^ Under aerobic conditions, they are completely degraded to water and carbon dioxide, while under anaerobic conditions, the microorganisms in soil, sewage, seawater, and lake water turn them into methane.^53,56^

**Figure 2:**
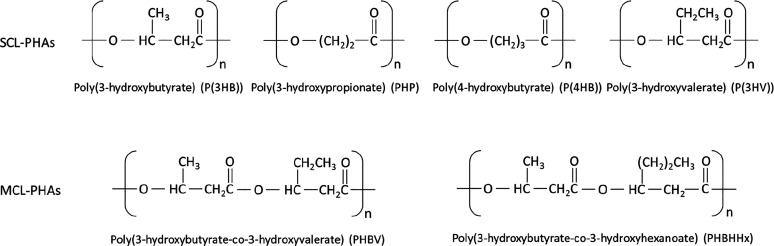
Chemical structures of some short- and medium-chain length PHAs.

Environmentally friendly polymers from the PHA group are considered the future of polymers. So far, over 150 different PHA monomers have been identified,^10,12,50,52−54^ and the molecular weight of PHA polymers is in the range of 50 000 to 1 000 000 Da.^4^ The chemical structure of polymers makes it possible to classify PHA polymers into two groups according to their chain length: short-chain length (SCL) or medium-chain length (MCL)^4,5,44,48−54^ (Figure 2).

Short-chain PHAs (SCL-PHAs) have 3 to 5 carbon atoms in the monomer, while medium-chain PHAs (MCL-PHAs) have 6–14 carbon atoms in their monomers.^4,50,51,53,54^ The chain length clearly defines the properties of PHA polymers. Short-chain PHAs are relatively brittle and have a high crystallinity and melting point. The exception in this subgroup of polymers is poly(4-hydroxybutyrate) (P(4HB)). However, medium-chain PHAs have elastomeric properties, including low melting point and low crystallinity. Furthermore, MCL-PHAs have a lower tensile strength value compared to SCL-PHAs. Obviously, the side-chain length as well as the presence and type of a functional group have a huge impact on the PHA properties.^48,51,54^ On the basis of the currently known PHA monomer units, these can be classified into homopolymers or heteropolymers. Homopolymers such as P(3HB) or poly(3-hydroxyoctanoate) (P(3HO)) contain a single repeating monomer unit. Conversely, heteropolymers from the PHA group contain more than one type of monomer in the chain, e.g., poly(3-hydroxyhexanoate-*co*-3-hydroxyoctanoate) (P(3HHx-*co*-3HO)) or (P(3HB-*co*-3HV)); see Figure 2.

## 3 Properties of Polyhydroxyalkanoate Polymers

PHA polymers are completely isotactic, hydrophobic, insoluble in water, and resistant to hydrolytic degradation and have piezoelectric properties. These materials have a much better resistance to UV degradation than polypropylene (PP). Their mechanical properties depend, as already mentioned, on their chemical structure.^50,51^ Nevertheless, they are generally more brittle and have a lesser elongation at break than PP or polyethylene (PE).^4,47,50,56^ In general, the properties of PHA polymers have a very wide scope, although their most important feature is their total biodegradability. The degradability of PHA materials is explained in more detail in Section 4.

The mechanical properties of PHAs are determined by monomer components, chain length and distance between side groups, and ester linkage.^54^ The first-discovered (Lemoigne, 1926) and best-known polymer from the PHA family is P(3HB).^51,52^ The parameters of the mechanical properties of P(3HB) are shown in Table 1. Compared to petroleum-based polymers (e.g., PP), it has a relatively high tensile strength and similar melting temperature. Pure P(3HB) is a semicrystalline polymer; hence, it is brittle and has a low elongation at break (4–5%). Due to the fact that the glass transition temperature (*T*_g_) of P(3HB) is close to room temperature, its elongation at break is significantly reduced.^54,61^ However, P(4HB) is a flexible material with around 1000% elongation at break and a much lower Young’s modulus (0.1–0.15 GPa) than P(3HB).^54,62^ Although these two polymers have the same number of carbon atoms in their chains, their mechanical properties are completely different. The main difference between them is the position of the methyl group in the chain (Figure 2), which affects the change in polymer crystallinity and changes both the 3D structure of the monomer molecule and the mechanical properties of the polymer.^54,61,62^

**Table 1:** Thermal and Mechanical Parameters of PHA Films Compared with PP, PS, and LDPE

samples	melting temperature (°C)	glass transition temperature (°C)	tensile strength (MPa)	elongation (%)	elastic modulus (GPa)	references
P(3HB)	175–180	4–9	40–45	4–5	3.5–3.8	(42, 52, 57, 58)
						
P(3HB-*co*-3% 3HV)	170		38		2.9	(53)
P(3HB-*co*-11% 3HV)	157	2	38	5	3.7	(57)
P(3HB-*co*-20% 3HV)	114–145	–5 to (−1)	20–32	27–50	0.8–1.9	(52, 53, 57)
P(3HB-*co*-28% 3HV)	102	–8	21	700	1.5	(57)
P(3HB-*co*-34% 3HV)	97	–9	18	970	1.2	(57)
						
P(3HB-*co*-3% 4HB)	166		28	45		(53, 59, 60)
P(3HB-*co*-10% 4HB)	159		24	242		(53, 59, 60)
P(3HB-*co*-64% 4HB)	50		17	591	30	(53, 60)
P(3HB-*co*-90% 4HB)	50		65	1080	0.1–0.15	(53, 54, 60)
						
P(3HB-*co*-10% 3HHx)	127	–1	21	400		(42, 58)
P(3HB-*co*-15% 3HHx)	115	0	23	760		(58)
P(3HB-*co*-17% 3HHx)	120	–2	20	850		(42, 58)
						
LDPE	130	–30	10	620	0.2	(52, 53, 57)
polystyrene (PS)	110	21	50		3.1	(42, 53)
polypropylene (PP)	170–176	(−10) to 45	34–38	400	1.7	(42, 52, 53)

Mixing PHB polymer with other PHA units, in various proportions, can improve the properties of the material, reducing processing temperature or brittleness.^54,61^ One of the most studied P(3HB)-based copolymers is P(3HB-*co*-3HV). An increase in the P(3HV) fraction in the copolymer reduces the melting point and significantly increases flexibility (Table 1).^53,57^ The increase in the molecular fraction of the HV monomer in P(3HB-*co*-3HV) led to a decrease in the melting point (*T*_m_) of the copolymer, from 175 °C to about 97 °C, at 34 mol % of HV. As the 3HV fraction increases, the copolymer becomes more flexible; i.e., elongation at break increases and the value of Young’s modulus decreases, as shown in Table 1. The combination of other monomers, e.g., 3HHx or 4HB, makes it possible to produce new copolymer compositions, whose properties can be changed and improved depending on their specific application.

**Figure 3:**
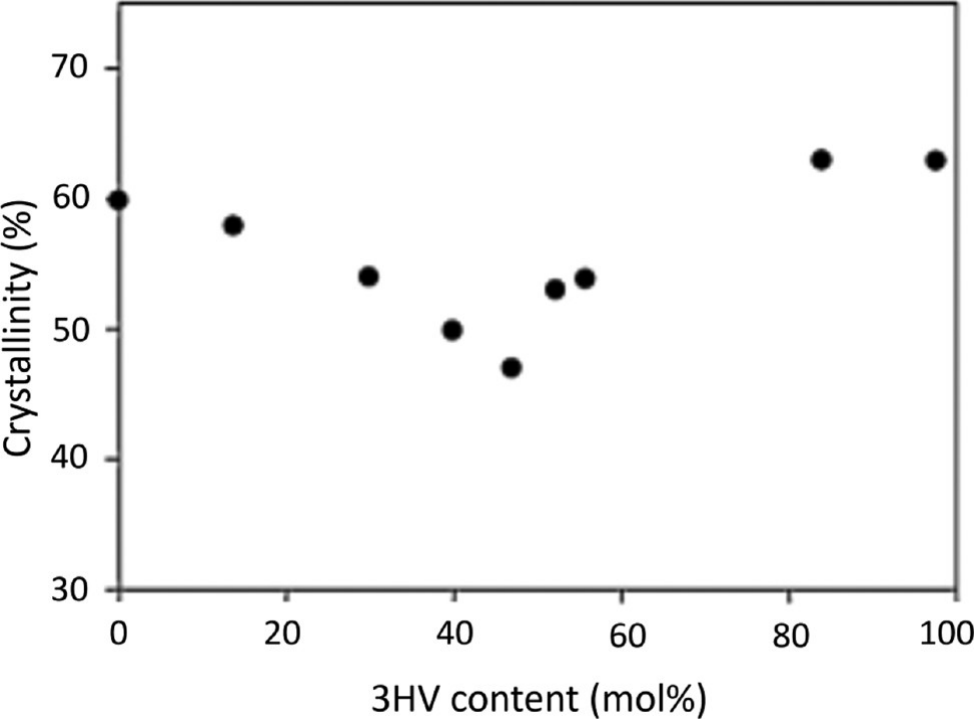
Effect of 3HV content in P(3HB-co-3HV) on the polymer crystallinity examined by wide-angle X-ray diffraction (WAXD). Reproduced from ref (68). Copyright 2001 American Chemical Society.

The crystallinity of PHA polymers is a phenomenon that has been studied widely.^50,52−54,61,63−72^ The different content of 3HB and 3HV monomers does not significantly affect the crystallinity of P(3HB-*co*-3HV). The degree of crystallinity ranges between 50% and 70% and is close to the degree of crystallinity of the P(3HB) homopolymer.^53,68,73^ Wang et al.^68^ observed that the crystallinity decreases slightly as the 3HV percentage increases in the copolymer; see Figure 3. Within 40–50 mol %, the lowest degree of crystallinity was observed, while above 50 mol %, crystallinity increased.

## 4 Degradation of PHA Polymers

One of the most important and the most attractive features of PHAs, compared to petroleum plastics, is their degradability. As mentioned earlier, PHA polymers degrade into water and carbon dioxide in aerobic conditions, while in soil, sewage, seawater, and lake water, microorganisms (under anaerobic conditions) turn them into methane.^4,51,74^ External factors, i.e., temperature, humidity, pH, and microbiological activity of the environment, have a huge impact on the rate of degradation.^4,53,74,75^

**Figure 4:**
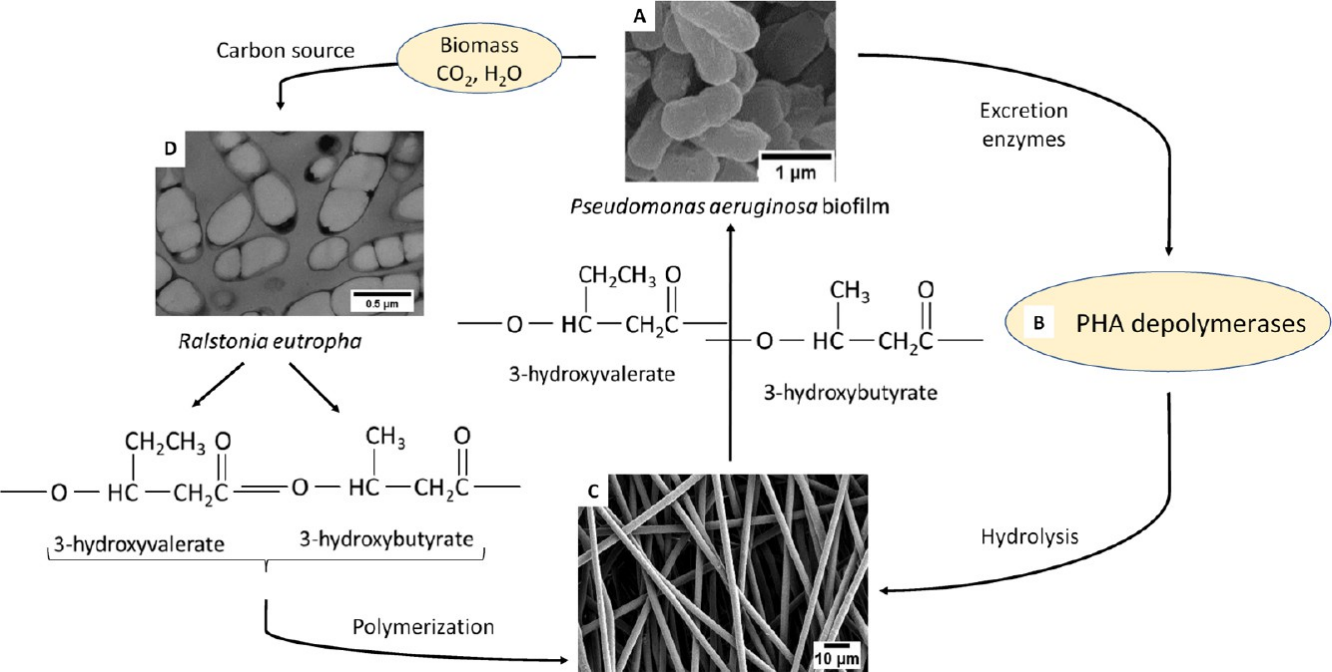
Schematics of the cycle of extracellular enzymatic degradation of PHBV. (A) SEM image of *Pseudomonas aeruginosa* biofilm, (B) PHA depolymerases (PHA-degrading enzymes, PHA e-depolymerases), (C) SEM micrograph of electrospun PHBV fibers, and (D) TEM micrograph of a thin section of recombinant *Ralstonia eutropha*. In the cycle, (A, B) microorganisms produce enzymes, (B, C) PHAs are hydrolyzed by PHA-degrading enzymes to monomers, (C, D) the further hydrolysis of the material and the hydrolyzed components are the source of carbon for the microorganisms; (A–D) hydrolyzed PHAs are used by microorganisms as the source of carbon and energy; (C, D) the components excreted by bacteria are chemically treated, polymerized, and produce materials based on PHA. Panel A reproduced with permission from ref (78). Copyright 2016 Mu, Tang, Liu, Sun, Wang, and Duan. Panel D reproduced with permission from ref (52). Copyright 2000 Elsevier.

Microorganisms produce enzymes that break the polymer down into molecular building blocks called hydroxy acids, which are used as a source of carbon for growth.^4,76,77^ The main enzyme for degradation of PHA and polymer-derived oligomers is PHA depolymerase. As a result of extracellular degradation, PHAs are hydrolyzed by PHA-degrading enzymes (PHA e-depolymerases) to monomers and oligomers.^51,76,77^ PHA depolymerases are enzymes excreted by bacteria and fungi.^51^Figure 4 shows the extracellular enzymatic degradation cycle. In the intracellular degradation mechanism, PHAs are hydrolyzed by intracellular depolymerases (PHA i-depolymerases).^51^

Jendrossek and Handrick^77^ demonstrated that the physical form of PHA is an important factor influencing the process of intra- and extracellular degradation. PHA found in bacteria is amorphous, while it is semicrystalline in the natural environment.^77^ Thus, in the case of PHA polymers, the factors affecting degradation are crystallinity and external factors.^70,77,79^ Polymers made of one type of monomer (e.g., PHB) degrade faster than their copolymers (e.g., PHBV).^4^ Volova et al.^80^ examined the degradation of four types of PHA using 35 bacterial isolates from 16 genera. Each of the PHA polymer types degraded when in contact with other bacteria, while all of them were degraded by *Streptomyces*.^80^ Feng et al.^81^ investigated the effect of enzymatic hydrolysis on P(3HB-*co*-3HV) with different contents of 3HV fractions and 3HB through the action of poly(3-hydroxyalkanoates) depolymerases isolated from *Ralstonia pickettii* T1 and *Acidovorax* sp. TP4. The results obtained in the research made it possible to discuss the mechanism of enzymatic degradation. It was shown that the degradation rate in the case of an action of both depolymerases increased with the increase in the content of 3HV. The maximum degradation rate was achieved with approximately 40% of 3HV, i.e., at a concentration that indicates the lowest degree of crystallinity,^68^ as shown in Figure 4. Moreover, it was found that the enzymatic degradation of PHBV is influenced by the composition of the copolymer and the contribution of individual fractions as well as by the structure of the solid polymer and the source of bacterial depolymerases.^81^ Saito and Doi^60^ conducted studies on the enzymatic degradation of P(3HB-*co*-4HB) with various proportions of copolymer components. These authors tested the biodegradability of materials by using either *Alcaligenes faecalis* hydroxybutyrate depolymerase or *Rhizopus delemer* lipase. In the reference sample, i.e., without the addition of the enzyme, no material degradation was observed. It was noticed that the acceleration of the degradation by PHB depolymerase varies, depending on the proportion of individual fractions in P(3HB-*co*-4HB) and thus on the crystallinity of the copolymer. Moreover, it was found that the PHB homopolymer is not degraded by lipase, but the copolymer degradation rate by lipases increased with the increase in the proportion of the 4HB fraction. Therefore, it can be concluded that P(3HB-*co*-4HB) materials are hydrolyzed by both lipase and depolymerase PHB.^60^ Doi et al.^82^ conducted an analysis and compared the enzymatic and hydrolytic degradation of the copolymers of P(3HB-*co*-4HB) and P(3HB-*co*-3HV). The enzymatic degradation was carried out in a solution at pH 7.5 and 37 °C containing P(3HB) depolymerase derived from *Alcaligenes faecalis*. It was noted that the rate of enzymatic degradation was much faster than that of the hydrolytic degradation. Moreover, as a result of enzymatic degradation, both the molecular weight of the copolymers and the weight of the samples decreased. It was found that, in the case of both hydrolytic and enzymatic degradation, the presence of 4HB units in polyesters increased the rate of material degradation.^82^ In the case of copolymers, the proportion of individual monomers affects the overall length of the segments and thus the mechanical properties of the copolymer.^83^ Chuah et al.^83^ investigated the biodegradability of the copolymers with varying proportions of 5-hydroxyvalerate (5HV). The 5HV homopolymer undergoes enzymatic degradation by lipases. It has been proved that the content of 5HV in the copolymer structure reduces the crystallinity and, consequently, supports the enzymatic degradation process.^83^

The form of PHBV, whether a film or fibers, affects the speed of the degradation process.^84^ The degradation of the film and electrospun fibers in simulated aerobic composting of municipal solid wastes was tested over a 100-day period. The degradation process of fibrous membranes was faster than in the case of the films: an event that can be explained by the larger specific surface area and high porosity of the material.^84^ Yuan et al.^28^ conducted a degradation test on PHBV fibers and keratin blends. Fibers were incubated in phosphate buffered saline (PBS) supplemented with PHB depolymerase (*Pseudomonas stutzeri* BM 190) or trypsin. As a result of enzymatic reactions using PHB depolymerase, fibers were seriously damaged, while in the trypsin solution, slight changes in the fiber morphology were observed, even after a degradation time twice as long.^28^ The phenomenon of degradation of the PHA group polymers has been widely studied, including hydrolytic and enzymatic degradation. Moreover, the biodegradation of PHAs is one of the key factors affecting the common waste disposal problem. Therefore, polymers from the PHA family are being used more and more widely in medicine.

## 5 Biocompatibility of PHA Polymers

**Figure 5:**
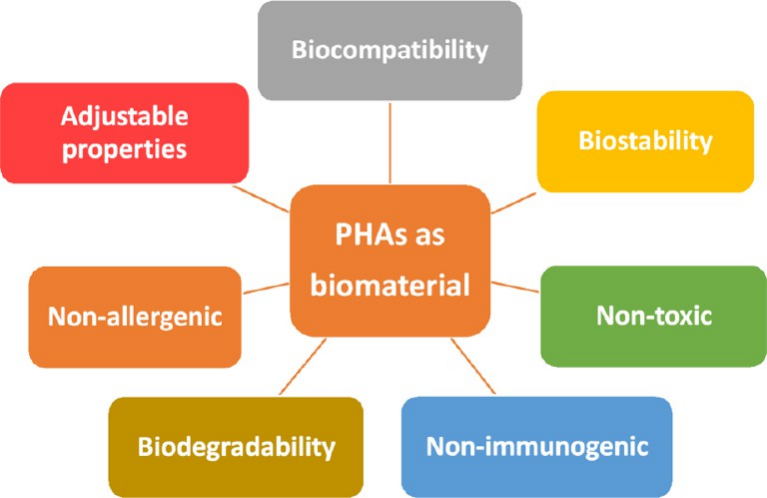
Most important properties of PHAs when used as biomaterial.

Biocompatibility is a key feature of both natural and synthetic polymers, which enables them to be used in the biomedical field. In addition to biocompatibility, biomaterials should have appropriate mechanical properties for their place of application, no toxic and allergenic degradation components, a controlled degree of degradation, and appropriate surface properties (see Figure 5). The biocompatibility of PHAs has been extensively investigated through *in vitro* studies using basic types of osteoblasts,^85−89^ fibroblasts,^89−94^ keratinocytes,^95−98^ chondrocytes,^99−103^ hepatocytes,^104,105^ or mesenchymal stem cells,^83,95,106−112^ thus confirming the biocompatibility and lack of cytotoxicity of PHBV materials.

*In vivo* biocompatibility is most often achieved by implanting materials into an animal body, while checking tissue responses and biodegradation phenomena.^102,111,113−117^ The high *in vivo* biocompatibility of PHA materials, especially the PHB polymer, can be demonstrated by the presence of 3-hydroxybutyrate, a degradation product that is naturally produced in the liver during the breakdown of long-chain fatty acids.^45,56,118^ The wound healing effect has been studied using materials in the PHA family. The tested materials had a different geometry, molecular weight, and/or as in the case of fibers, diameter. Tissue reactions to PHBV fibers are relatively low, thus indicating a high biocompatibility. Kuppan et al.^113^ compared the use of electrospun PHBV fibers in skin wound healing. The wound healing in the rat model was obtained using pure PHBV fibers and the angiogenesis factor (R-Spondin 1) loaded into PHBV fibers. It has been found that the presence of an angiogenesis-promoting factor greatly reduces the wound size.^113^ The biodegradation of the implanted material is closely related to the *in vivo* biocompatibility. Freier et al.^114^ developed resorbable gastrointestinal patches of solution-cast film of PHB, PLLA, and PHB modified to atactic PHB to accelerate degradation. The blend of PHB and atactic PHB was selected from test materials to repair an intestinal defect in Wistar rats. After implantation, a slight residual material was observed in only one rat out of a group of four. A high resistance of the material to intestinal secretions, optimal degradation time, and healing of intestinal wounds were seen in all cases.^114^ Inflammatory reactions are key parameters of *in vivo* tests, used to ascertain the biocompatibility of materials. In their research, Qu et al.^119^ implanted pure poly(3-hydroxybutyrate-*co*-3-hydroxyhexanoate) (P(HB-*co*-HHx)), PHB, PLA materials, and blends of P(HB-*co*-HHx) and poly(ethylene glycol) (PEG) subcutaneously into rabbits. The best results were obtained for P(HB-*co*-HHx), where the tissue reaction was characterized by the lowest extent of fibrosis and no inflammatory cells.^119^

The materials used for all biomedical applications are characterized by a number of optimal properties mentioned earlier (Figure 5). Various techniques allow one to obtain products with desirable properties, e.g., adequate strength and porosity and controlled degradation over a specific period of time.^120^ There are many classic techniques for the production of biomaterials, including lyophilization, phase separation, and gas formation; however, these techniques are inaccurate and do not allow for the control of the internal structure and the production of a diversified structure of the material.^121^ Modern rapid prototyping techniques (3D printing, selective laser sintering, stereolithography, fused deposition modeling) allow one to easily produce architecturally diverse scaffolds.^121^ Rapid prototyping methods are characterized by product repeatability, but a significant limitation is the small number of materials that can be processed with these methods.^121^ However, an interesting approach that is gaining popularity is electrospinning of polymer fibers. Various polymers can be used to obtain fibers; the process gives many opportunities to optimize the properties of the obtained fibers, and additionally, nanofiber scaffolds reflect the way of building tissues. The electrospinning of PHA materials is explained in more detail in the next section.

## 6 Electrospinning of PHA Polymers

**Figure 6:**
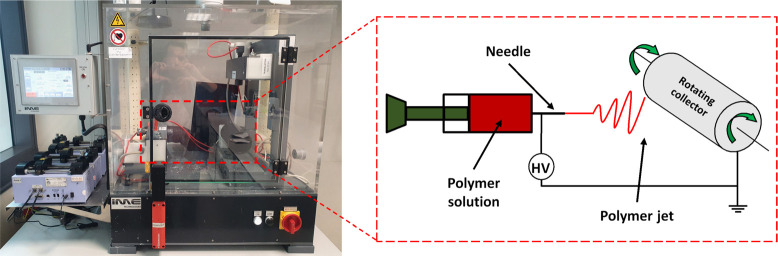
(A) Standard electrospinning setup with climate control chamber; (B) schematic of the electrospinning system with the syringe pump to deliver the polymer solution, needle where the HV is applied, and the rotating collector in which the system is grounded and the fibers are deposited.

Electrospinning has been known for many years and is an incredibly widespread method for the production of fibers of various sizes. This technique makes it possible to produce nano- and microscopic fibers.^122^ The electrospun fibers have an extremely high porosity index, even up to 99%, and a large specific surface area. In addition, these fibers are characterized by a considerable length and a small cross-section, the diameter of which is approximately 100 times smaller than its length.^54,123−129^ Electrospinning is the process of manufacturing fibers from a melt polymer or polymer solution. Although electrospinning can be implemented fairly easily and cheaply, it is a complex process that depends on several parameters.^130^ There are many reports on the electrospinning process in the literature.^123−125,128,129,131,132^ Briefly, the electrospinning setup, as shown in Figure 6, consists of a polymer pump, collector, nozzle with needle, high-voltage source, and alternatively a climate control chamber. The polymer solution is pushed, using the syringe pump, through a metal needle (nozzle) to which a high voltage is applied. A potential difference is created between the needle and the collector. The electric field causes the polymer jet to be dynamically drawn, while solvent evaporates and solid fibers are produced.^123,124,126,133^ Numerous experimental studies confirm that the morphology of the fibers obtained depends mainly on the process parameters as well as on the composition of the polymer solution,^54,123,126,131^ applied voltage polarity,^123,125−127,133,134^ flow rate of the polymer,^54,123,126,131^ humidity^54,123,133,135^ and temperature during the process,^54,123,133^ and distance between the needle tip and the collector.^123,131,133^ Moreover, some changes in the electrospinning system, in particular the spinning needle system, make it possible to obtain core–shell,^136^ side-by-side,^137^ hollow, twisted, or multilayer structures.^123,125−127^ The electrospun fibers find many applications in the medical field, such as protective face masks,^1,2^ tissue engineering,^25,138^ especially for bone^139^ with controlled fibers’ surface potential^140,141^ and geometry,^142,143^ wound dressing, vascular grafts,^144^ heart valves,^145^ skin patches,^146−148^ sensors,^149^ and many more.^150−152^

The electrospinning of polymer fibers can be modified, as already mentioned, due to the possibility of adjusting numerous parameters. Extensive research on changes in the electrospinning parameters of PHBV polymer fibers and their impact on morphology was studied by Tong and Wang. The influence of voltage polarity and the effects caused by changes in the polymer solution concentration, polymer flow rate, distance from the collector, and even the diameter of the needle used were verified.^153−156^ Additionally, the effect of the fiber alignment on the mechanical properties was analyzed.^86,157^ The conducted research also shows that, with the increase of the needle diameter, larger fibers were obtained for both the positive and negative voltage polarity.^153,155,156^ The change in polymer flow rate did not significantly affect the average fiber diameter,^153,155^ but as the distance between the needle and the collector increased, the size of the fibers decreased.^153,155,156^ On the other hand, an interesting phenomenon was observed in the case of fibers produced with variable voltage polarity. In the case of the positive potential, larger fibers were obtained with the increase of positive voltage polarity (from +16 to 25 kV), while at very low negative potentials (−25 kV), the diameter of the fibers was 3 times lower than for the same potential with reversed polarity.^155^ Yoon et al.^158^ presented the effect of changing the polymer solution concentration on the morphology of the obtained fibers. It was noted that the lower polymer concentration (16 wt %) resulted in a bead structure, while at the higher (28 wt %) polymer concentration, no beads were observed.^158^ The obtainment of a stable polymer solution jet and a reproducible morphology and size of the fibers requires the adjustments for PHBV electrospun fibers, which are summarized in Table 2. The parameters for the electrospinning of the polymers can be varied to obtain different fiber sizes from nano up to micro.^159,160^ Electrospinning allows one to obtain fibers with a desire diameter in a controlled way; however, it is still challenging to produce fibrous structures with larger pores and spaces between fibers. Simonet et al.^161^ proposed a cryo-electrospinning model using dry ice to cool the collector under conditions of increased humidity. Ice crystals are deposited on the collector surface, which serves as a porosity template for the deposited polymer fibers. It was shown that nontemperature electrospinning allowed the porosity to increase up to 4 times. Ice crystal formation on the collector occurred at 30% humidity.^161^ The diversity of electrospinning makes it possible to match and obtain the desired fibers that gives the wide range of application for electrospun membranes and scaffolds, especially in biomedicine.

**Table 2:** Parameters (Solvents, Concentration of Polymer Solution, Voltage Polarity, Polymer Flow Rate, Distance to Collector, Temperature, and Humidity) of the Electrospinning Process for PHBV Electrospun Fibers

	HV contents (mol %)	solvents	concentrations (%)	voltage polarity (kV)	polymer flow rates (mL/h)	distance to collector (cm)	temperature (°C)/humidity (%)	fiber diameters (μm)	references
PHBV	3	chloroform	16–28	15	3.0	15			(158)
*M*_w_ = 680 000 g/mol									
PHBV	3	chloroform, DMF		15	3.0	15		∼1.0	(107)
*M*_w_ = 300 000 g/mol									
PHBV		chloroform	4–16	15–25	0.12–0.50	15–30	25–35		(162)
*M*_w_ = 1 000 000 g/mol									
PHBV	2	chloroform, DMF	8	17	6.0	20	25/40	2.6–2.8	(89, 92)
*M*_w_ = 450 000 g/mol									
PHBV	3	TFE	5–25	5–35	1.0–9.0	5–30		1.0–6.0	(155)
*M*_w_ = 310 000 g/mol									
PHBV		DCM, DMF	15	10	0.18	12		∼0.75	(113)
*M*_w_ = 450 000 g/mol									
PHBV	3	HFIP	8	14	1.0	18		0.30–0.45	(163)
PHBV	12	chloroform, TFE	16	25	1.0	15	25	0.40–0.80	(94)
PHBV	3	HFIP	8		1.0	12		0.205–0.266, 0.386–0.472	(164)
PHBV	3	chloroform	3	20	1.0	15		∼0.75	(165)
PHBV blends									
PHBV/HA composite	2	chloroform, DMF	8	17	6.0	20	25/40	∼2.92/∼3.76	(166)
PHBV/CFO composite	3	chloroform	10	20	1.0–2.0	15–20		∼4.4/1.4–1.7	(85, 87)
PHBV/gelatin blend	5	TFE	2–8	7	2.0	12		0.4–1.0	(91)
PHBV/chitosan blend	12	TFA/HFIP	10	22	0.48	14		0.20–0.40	(167)
PHBV/PLGA blend	5	chloroform, DMF	5–15	15–25	0.6–1.8	15–25		∼1.50	(168)
PHBV/silk fibroin	3	HFIP	10	20	1.0	15		0.28–0.38	(90)
PHBV/PEO blend	12	chloroform	20	12–15	0.4–0.5	10–12		2.60–2.80	(169)
PHBV/HA composite	2	chloroform	1	10–15	2.0–5.0	15		9.27–18.03	(170)
PHBV/PLA/PGS blend	5	chloroform, DMF	5	18	1.8	24		1.10–1.25	(171)
PHBV blends	5	HFIP	6	7–12	1.0–2.0	12–22		0.495–0.815	(172)
PHBV/CeO_2_ composite		chloroform, DMF	20	18	2.5	10		1.87–2.85	(96)
PHBV/PCL blend	12	chloroform	23, 10	7	2.0	5		∼7.17	(106)

## 7 Biomedical Application of PHBV Electrospun Fibers

The interest in electrospun fibers is constantly growing. The variety of the fibers obtained, size scale, and changes in morphology or porosity of nonwovens are just a few known characteristics that make the use of these fibers suitable in many fields of science and industry. However, the greatest interest in electrospun fibers comes from the biomaterials and tissue engineering fields. PHBV is more widely investigated and used in medicine than PHB due to its lower degree of crystallinity. It is known that heteropolymers degrade faster in the human body than homopolymers, as shown in Figure 3; the crystallinity of PHB is about 60%, and with the addition of the 3HV component, the crystallinity falls below even 50%.

**Figure 7:**
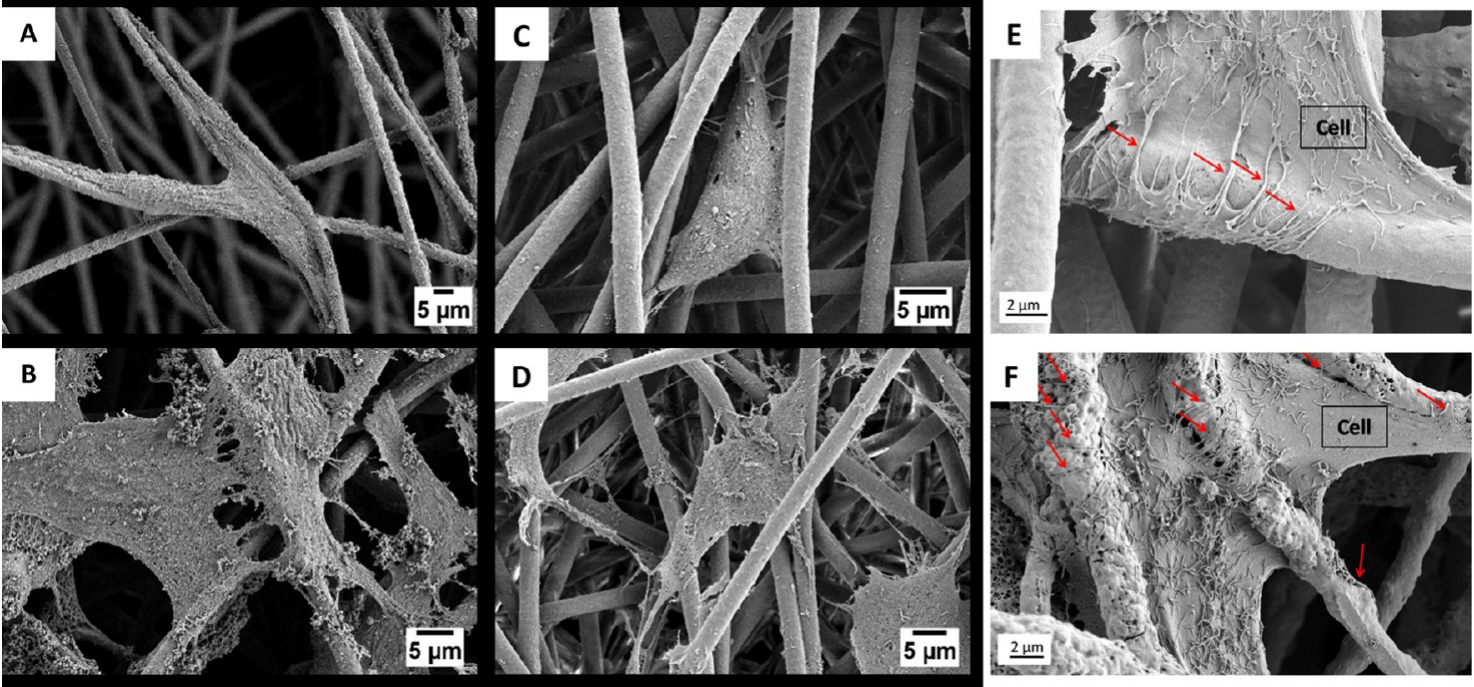
SEM micrograph showing (A, B) osteoblasts and (C, D)fibroblasts interaction with fibers after (A, C) 3 days and (B, D) 7 days; (E) cell morphology after 7 days of cell culture on PHBV fibers and (F) PHBV + HA fibers; red arrows indicate filopodia interacting with fibers. Panels A–D reproduced with permission from ref (89). Copyright 2020 Elsevier. Panels E and F reproduced with permission from ref (166). Copyright 2021 Karbowniczek, Kaniuk, Berniak, Gruszczyński, and Stachewicz.

There are many references to the medical applications of PHBV electrospun fibers, which can be broken down into the areas mentioned above, but it seems more important to evaluate their properties and modifications in order to achieve the desired features and functions in the human body. *In vitro* tests using PHBV material confirm its biocompatibility. The contact with the cells tested on the material in the form of electrospun fibers and flat PHBV films confirms a high compliance, while the fiber–cell interaction promotes cell migration and proliferation.^89^Figure 7 shows the interaction of osteoblasts and fibroblasts with PHBV fibers.^89,166^

**Figure 8:**
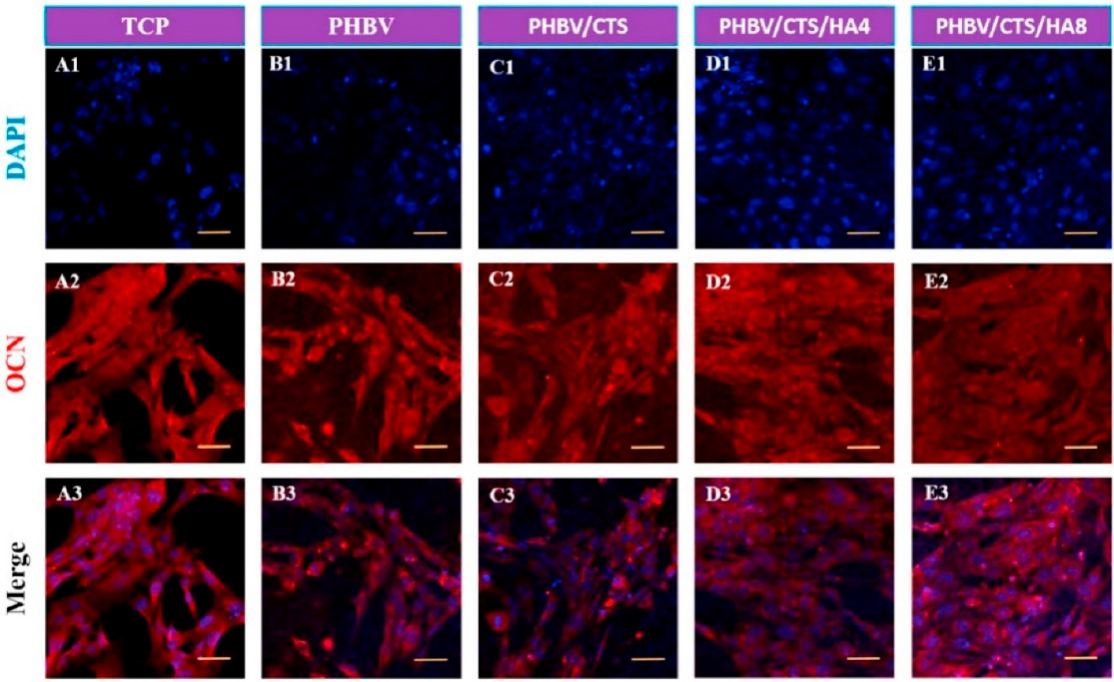
Osteocalcin expression by hFOB cells after 5 days of culture on TCP, PHBV, PHBV/CTS, PHBV/CTS/HA4, and PHBV/CTS/HA8,; pictures taken with 20× magnification; scale bar on images represents 100 μm. Reproduced with permission from ref (173). Copyright 2015 Elsevier.

When the PHBV fibers are modified, structures can be obtained that support the anchoring of cells and, thus, the regeneration of bone tissue. The fibrous PHBV scaffolds enriched with inorganic HA particles were seen to support the anchoring and attachment of osteoblasts (see Figure 7E,F).^166^ It has been shown that the inclusion of HA particles in the fiber structure increases cell proliferation. These studies confirmed that the combination of organic and inorganic materials produces a hybrid material, the properties of which can support bone regeneration.^166^ Zhang et al.^173^ used another approach to support the regeneration of bone tissue: a scaffold based on PHBV, chitosan, and HA particles. Calcium deposition in HA-enriched scaffolds, as confirmed by EDX analysis, may reflect the bone mineralization process. The proliferation and activity of ALP on day 20 significantly exceeded the results obtained for PHBV fibers and even the control. The expression level of osteocalcin (OCN), a bone-building protein produced only by osteoblasts, has been investigated. Figure 8 shows the results after 5 days of culture human fetal osteoblast (hFOB) cells. Increased levels of OCN expression and a more homogeneous distribution of cells on chitosan and HA particle scaffolds were observed. It was found that the potential resulting from the multicomponent scaffold can support bone tissue regeneration and reconstruction. Chitosan ensures cell migration, while HA supports the mineralization process.^173^

Nanofiber mats made of PHBV and silk fibroin subjected to a plasma (oxygen and nitrogen) treatment to increase the surface wettability of the materials were investigated by Unalan et al.^165^ It was found that the plasma treatment did not affect the morphology and size of the obtained fibers. The improved biocompatibility was ensured by the addition of silk fibroin, while the additional plasma treatment increased the hydrophilicity of the surface. Moreover, both the addition of silk fibroin and the plasma oxygen treatment proved to increase mineralization. A similar level of ALP activity was observed in all samples, but the most promising material, thanks to its hydrophilic properties and improved biocompatibility, turned out to be a material made of a mixture of PHBV fibers and silk fibroin modified by a nitrogen plasma treatment.^165^ Another example of a three-component material is a scaffold based on PHBV fibers with the addition of silk fibroins and HA nanoparticles.^170^ The presence of HA supported the mineralization process, which in turn supported the adhesion and proliferation of osteoblasts. The protein derived from silk fibroins made it possible to significantly mimic the structure of the natural ECM. On the basis of the *in vitro* tests conducted, high biocompatibility and biocompatibility of the three-component fibrous scaffolds were found.^170^ Various substances of the active proteins are of great importance in supporting the regeneration of bone tissue, i.e., collagen or poly-l-lysine. Kouhi et al.^163^ compared, for evaluation and optimization purposes, the physical and chemical properties of PHBV fibrous scaffolds in regulating cellular response. Poly-l-lysine (PLL) was incorporated into the scaffolding system by two methods: (i) surface immobilization of PLL based on covalent bonding and (ii) electrospinning of a polymer blend with the addition of PLL. *In vitro* studies using human fetal osteoblast (hFob) cells confirmed that the presence of PLL and especially the use of PLL in a blend increase proliferation and ALP activity and support bone protein expression and mineralization. These studies showed that, in order to improve the interactions of cells with the scaffold, it is extremely important to assess the ECM structure and function, which can be achieved by adding active protein substances.^163^ It has been shown that electrospun scaffolds, thanks to their structure, topography, and imitation of the natural ECM, can be used in the regeneration of bone tissue. The stimulation possibilities of materials increase the inclusion of cells within the material, thus promoting cell adhesion and proliferation. The optimization of the electrospinning process to enhance material properties in order to differentiate mesenchymal stem cells is described in detail by Wang et al.^174^

**Figure 9:**
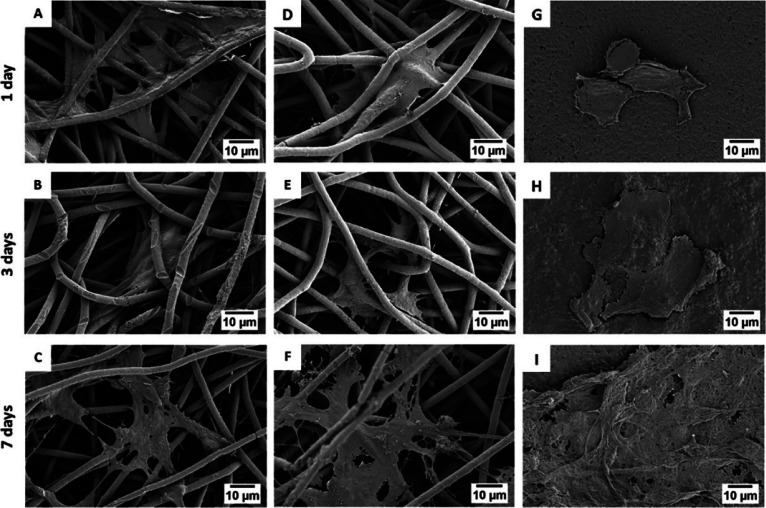
SEM micrograph of fibroblast attachment after 1, 3, and 7 days to (A–F) electrospun PHBV fibers and (G–I) PHBV films. Reproduced with permission from ref (92). Copyright 2021 Elsevier.

Another issue worth describing is the production and use of the skin model or wound treatment system using polymer fibers. Research has proven that the materials used to replicate, heal, and match the properties and functions of the skin can be jelly like or droopy substances, elastomers, or fibers.^175^ In the case of skin treatment, it is worth noting that the matrix for skin treatment should contain, for example, keratin or other fibrous proteins derived from epithelial cells or hair and nails.^28,172^ Fibroblasts, as the most numerous cells in connective tissue, are themselves able to produce collagen, and the addition of collagen significantly affects their activity. The activity and proliferation of fibroblasts (see Figure 9) has been the subject of many published studies.^90,92,113^ Both in electrospun fibers and in flat films, there was a high cell viability, indicating the possibility of a medical use for PHBV-based materials.^92,113^

**Figure 10:**
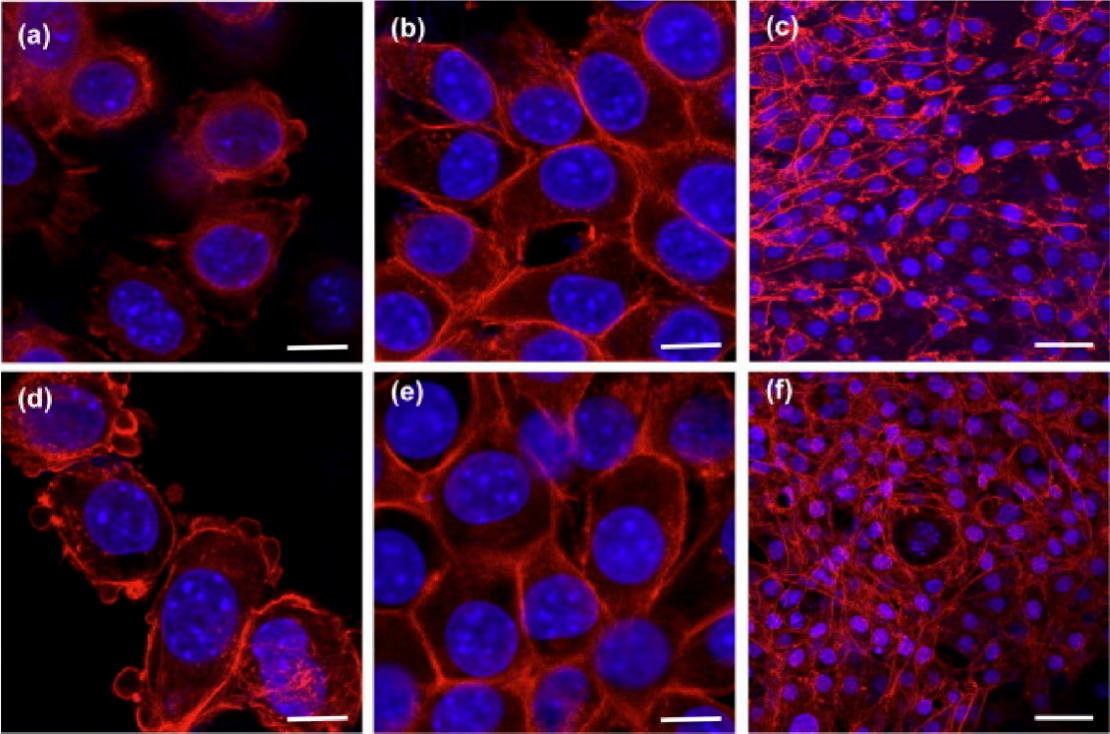
CLSM images of L929 fibroblasts on PHBV/chitosan (2:3, a–c; 4:1, d–f) for (a, d) 12 h, (b, e) 2 days, and (c, f) 7 days. F-actin (red) stained with Alexafluor 546 conjugated to phalloidin and nucleus (blue), with DAPI. Scale bar on (a, b, d, e) represents 10 μm and on (c, f), 50 μm. Reproduced with permission from ref (167). Copyright 2012 Elsevier.

Research has confirmed that electrospun PHBV mats modified with the addition of keratin can support regeneration and wound healing.^28^ An interesting issue described by Wang et al.^172^ is that of the study of the effects of adding keratin, gelatin, and collagen to the PHBV polymer solution on the development of fibroblasts. The blend of collagen fibers showed the greatest adhesion and proliferation, but the fibers with keratin also produced satisfactory results.^172^ In the literature, there are also reports on hybrid nonwovens with chitosan^167^ or curcumin^176^ used for skin regeneration. The weight fraction of chitosan in the PHBV solution mixture appeared to be very important in the research conducted. *In vitro* studies with the use of fibroblasts on the scaffolds made of a mixture with different proportions of PHBV and chitosan showed that in both cases there was an increase in proliferation during cell culture. *In vivo* studies carried out on male Wistar rats also showed that a 25% share of chitosan in hybrid fibrosis significantly affects the wound healing process (Figure 10).^167^ Curcumin-loaded nanofibers showed no cytotoxicity, and the viability of fibroblasts increased with increasing amounts of curcumin added.^176^ These examples confirm that the modifications of the spinning solution (PHBV/chitosan blend) and solid form additives (PHBV with curcumin) are very important; they improve the biological properties of the material, thus confirming their use in skin treatments.

**Figure 11:**
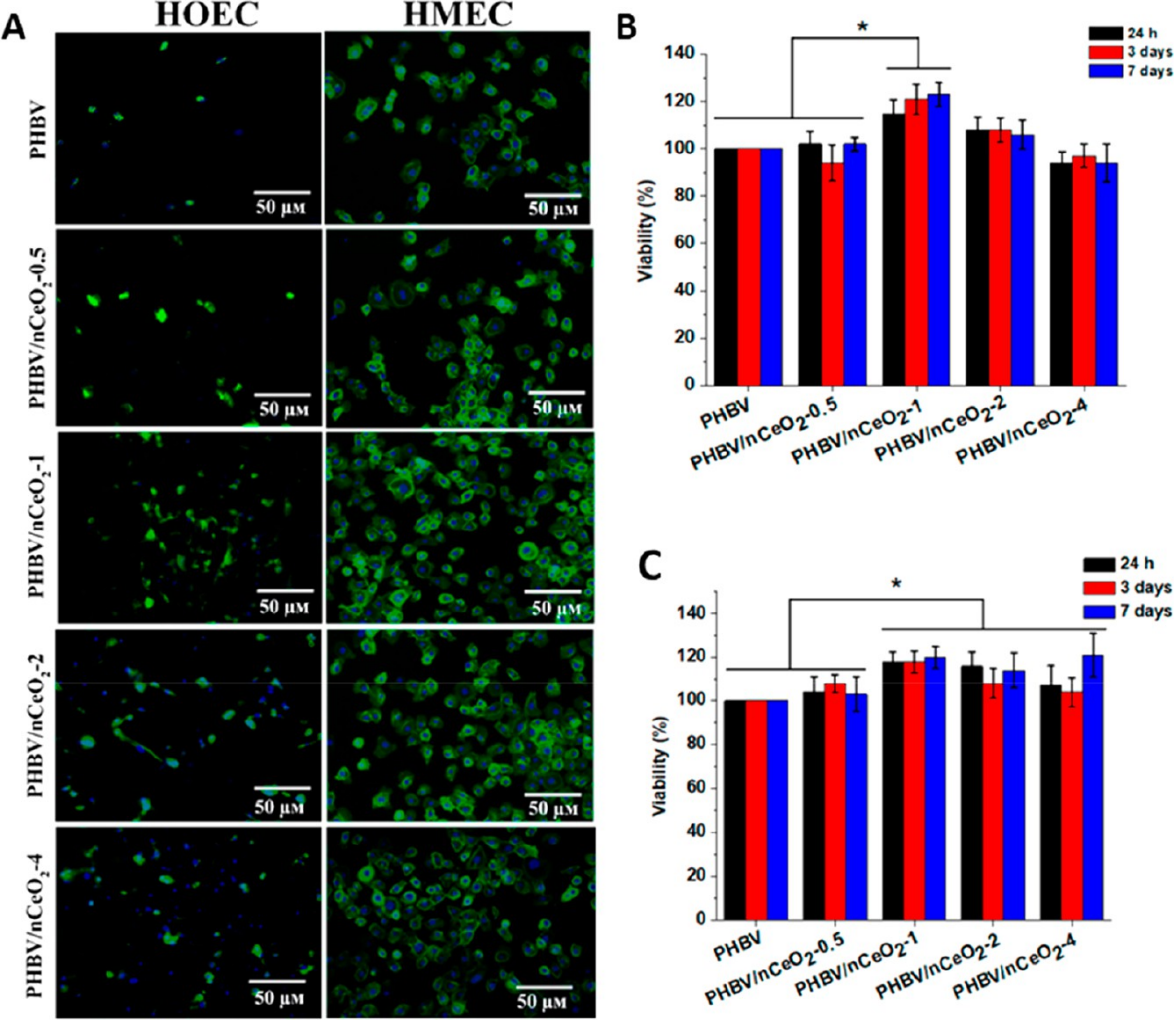
Adhesion of HOEC and HMEC to the membranes. (A) CLSM images after 3 days of cell culture by DAPI–phalloidin staining on the PHBV/*n*CeO_2_ membranes, (B) cell viability (MTT assay) on HOEC, and (C) HMEC on the PHBV and PHBV/*n*CeO_2_ membranes. Reproduced from ref (96). Copyright 2020 American Chemical Society.

The high cell proliferation and the angiogenesis process are very important in the treatment of chronic and difficult-to-heal wounds. Augustine et al.^96^ presented a model of a PHBV dressing with the addition of cerium oxide (*n*CrO_2_) nanoparticles. Membranes with *n*CrO_2_ (1 wt %) promoted the proliferation and adhesion of two cell types: human oral epithelial cells (HOEC) and human mammary epithelial cells (HMEC) (see Figure 11). The addition of *n*CrO_2_ was seen to influence both the formation of blood vessels and the faster wound healing in diabetic rats. These studies confirmed the potential use of PHBV dressings with the addition of *n*CrO_2_ nanoparticles.^96^

In the treatment and regeneration of skin and wounds, antibacterial properties are important. One of the examined examples of the membrane was a blend of electrospun fibers made of PHBV and poly(ethylene oxide) (PEO) with the addition of zinc oxide (ZnO) nanoparticles.^177^ The antibacterial properties of the composites were tested using bacteria that cause wound infections: *Staphylococcus aureus* and *Pseudomonas aeruginosa*. A more effective antibacterial activity was observed with an increased ZnO concentration. In addition, *in vitro* studies using the mammalian fibroblast L929 cell line confirmed the lack of cytotoxicity of the tested composites. The research confirmed the antibacterial properties of the PHBV and PEO fiber blend with ZnO nanoparticles as well as the enormous potential of antibacterial modified materials for wound healing treatments.^177^

**Figure 12:**
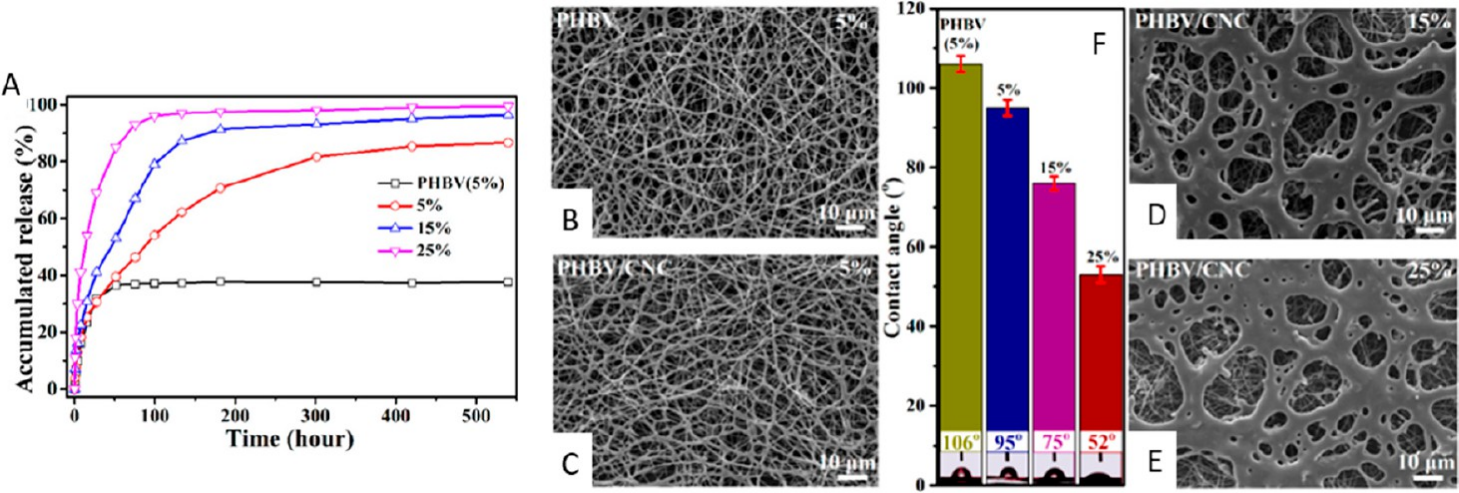
(A) Accumulated drug release from composite nanofibrous membranes with 6 wt % CNC loading. (B–D) FE-SEM images and (F) contact angles for drug-loaded PHBV nanofibers and composite nanofibrous membranes with different drug loadings (B, 5%; C, 5%; D, 15%; E, 25%). Reproduced from ref (36). Copyright 2017 American Chemical Society.

Drug delivery is another important aspect investigated in the use of PHBV electrospun fibers. Cheng et al.^36^ investigated drug release (tetracycline hydrochloride) from nanofiber membranes modified with cellulose nanocrystals (CNCs). The addition of CNCs improved the mechanical properties of these membranes and changed the nature of the material from hydrophobic to hydrophilic. A composite material with 6 wt % CNC content was loaded with 5–25% of the drug. An initial burst of drug release followed by a slow release was observed. The maximum drug release efficiency in PHBV membranes with CNCs ranged from 80% to 99%, while the drug delivery rate from unmodified PHBV fibers was less than 40% (see Figure 12). The results of this research showed the enormous potential of PHBV fibers in a long-term drug delivery system. Another study investigated the antibacterial properties and also the drug release (tetracycline hydrochloride) of an electrospun drug-loaded blend of PHBV and PLA. Antibacterial properties were investigated using *Escherichia coli* and *Staphylococcus aureus*. Drug-adsorbed nonwovens showed satisfactory antimicrobial properties, suggesting the potential use of drug release membranes in skin regeneration.^178^

PHBV fibers show a great potential for advanced applications in tissue engineering such as cardiac patches^171^ or grafts for nerve tissue engineering.^164,168,179^ The heart muscle is not able to regenerate when damaged. The heart reconstruction after a heart attack consists of removing a fragment of the coronary artery and implementing a cardiac patch.^171^ Polymer fibers from a mixture of PHBV, PLLA, P(d,l-LA), and poly(glycerol sebacate) (PGS) were aligned to mimic the fibers in the heart muscle. The targeted fibers supported the targeting of mesenchymal stem cells (MSCs). The cells also showed an activity inside the mats, penetrating the spaces among the fibers. In addition, macroporous tubes were produced to provide nutrients. The method of producing a multilayer patch with a structure similar to the heart muscle is a major step forward toward the treatment of postinfarction injuries and other defects in the heart muscle.^171^ Aligned electrospun fibers can also be used in the engineering of nerve tissue.^164^ The reconstruction of nervous tissue is one of the main problems in current regenerative medicine.^179^ There are two main approaches to protecting and restoring the nervous system. The protection issue focuses on dosing drugs in order to stimulate and restore the continuity of the nervous tissue. Regeneration is based on supporting new axons and the reconstruction of nerve connections with the use of a support, e.g., electrospun fibers.^179^ For this purpose, aligned PHBV fibers and blends of PHBV and collagen fibers were tested. The biocompatibility and the effect of fiber directionality on cell development were investigated using the PC12 nerve cell line. The fiber direction affected the proliferation of nerve cells, making it possible to elongate cells in the direction of the fibers, while the addition of collagen improved their mechanical properties. This research confirmed the potential of aligned nonwovens in tissue regeneration based on directional cell growth.^164^ Electrospun fibers of PHBV, PLLA, P(d,l-LA), and PLGA polymer blends were used to create a model for nerve reconstruction. The electrospun fibers in the PHBV and PLGA blend with an aligned structure were used as the core, and a mixture of three polymers was used as the protective tube. Polymers were selected to ensure that the protective tube degraded more slowly than the core, which should be completely regenerated during this time. The structure and properties of the nerve conduit produced indicate that it is a good candidate for further research on nerve regeneration.^168^

The examples offered for the use of PHBV fibers or their modifications emphasize their importance and possibilities for medical applications. A benefit of the diverse morphology and topography of electrospun fibers is the possibility that they offer a wide range of products for use in the treatment and reconstruction of bone tissue or skin as well as in more advanced applications for the regeneration of damage to the nervous system. The widely studied system for long-term and controlled drug delivery can also be represented by the use of nanofiber polymer membranes. In summary, electrospun PHBV fibers are a promising alternative for applications in tissue engineering.

## 8 Application of Other PHA Polymers

The industry involved in the production and processing of polymers still struggles with two main problems: the use of harmful petroleum-based chemicals and the fate of polymeric materials, i.e., waste management.^180^ Biodegradable polymers can help with the implementation of these guidelines. Products made of microbial PHAs, as examples of biodegradable polymers, can be widely used in polymer processing, and their properties (see Section 4) support a rational waste management. The commercialization of polymers from the PHA group dates back to the second half of the 20th century. In 1970, the PHBV polymer was commercialized under the trade name Biopol by Zeneca BioProducts (Bellingham, UK).^63,74^ For several years, this company was the only industrial-scale manufacturer of P(3HB) and P(3HB-*co*-3HV).^74^ PHA has numerous applications: many of them are closely related to medicine, e.g., surgical sutures, wound dressings, or bone plates. However, food packaging is so far the principle industrial application of PHA polymers.^181−188^

### 8.1 Biomedical Application of Other PHA Polymers

**Figure 13:**
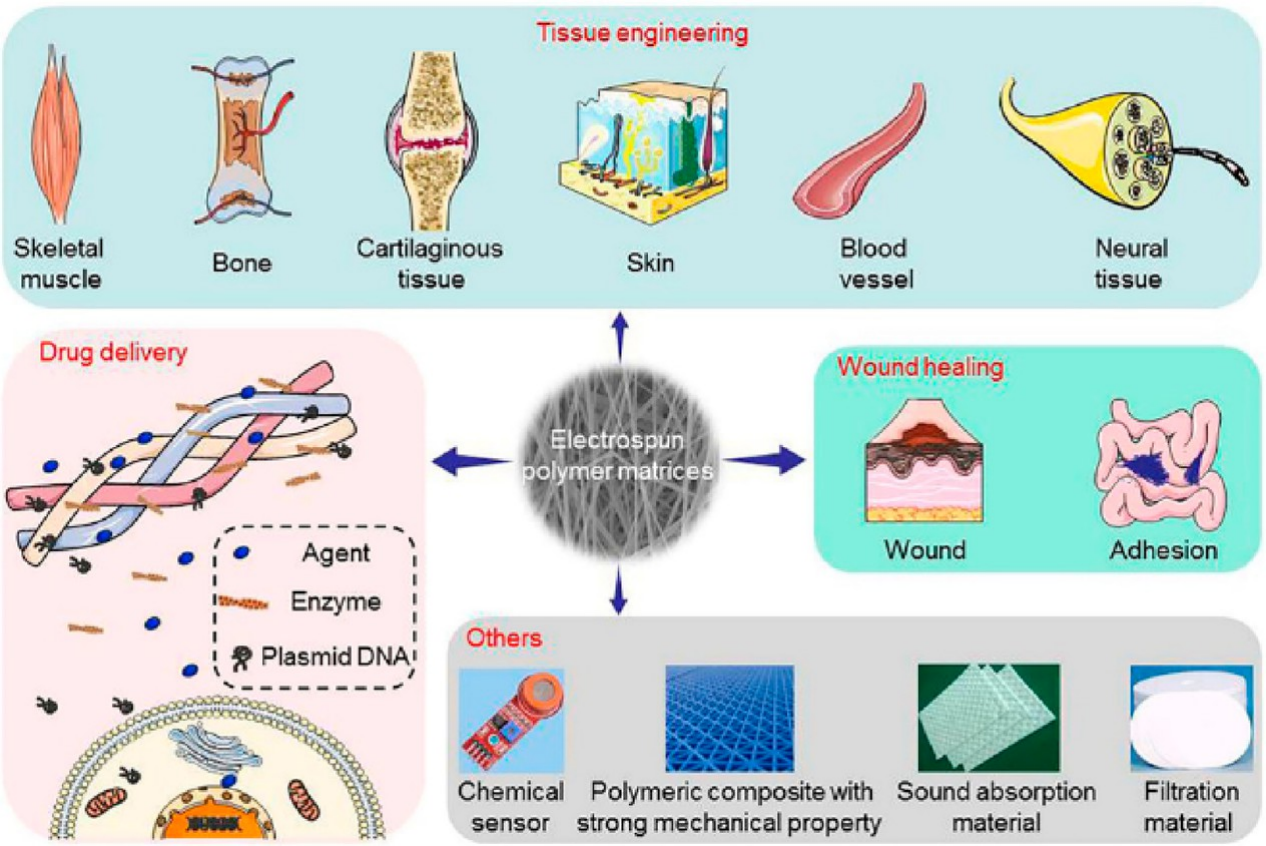
Biomedical applications of PHAs. Reproduced with permission from ref (123). Copyright 2019 Elsevier.

The largest, best-known, and most continuously improved applications using PHA are active and inactive medical products. The most important characteristic of a material for medical applications is biodegradability, which is discussed in Section 5. Polymers from the PHA group are biocompatible, because their degradation product (3-hydroxybutyric acid) is a product of cell metabolism and is present in the human blood in concentrations of 0.3–1.3 mM.^70,189^ As mentioned earlier for the degradation of the films cast from a solution of PHB, some modifications of PHB and PLLA were used to develop gastrointestinal patches.^114^ The material used to heal defects in gastrointestinal areas, in addition to showing sealing features and a controlled degree of degradation, must support the tissue, be flexible and saturable, and most importantly, be resistant to gastrointestinal secretions and digestive enzymes. Moreover, an important factor in the application of intestinal plasters is their surface morphology. This aspect is very important in most tissue engineering materials. On the one hand, the material should be porous and support cell adhesion and the healing of intestinal defects, while on the other hand, it should be smooth enough to prevent cell attachment and fibrinous adhesion.^190^ The smooth surface of patches was produced by the dip method from a chloroform solution. The porous structure was obtained by adding sodium chloride to the solution, and the material, after washing away the salt, produced a porous film surface. PHB-based materials showed optimal mechanical and degradation properties for supporting cavities and repairing gastrointestinal tissues.^114^ Other studies on PHA polymers focused on comparing the biocompatibility and biodegradability of films with PHB and PHBV as a cell culture study.^185^ The surface properties were assessed, and the strength before and after sterilization was compared in both types of polymer films. Fibroblasts grown on substrates showed a high degree of adhesion in both cases. Samples showed no cytotoxicity and, therefore, it can be concluded that these materials are useful as a cell surface for *in vitro* tests.^185^ Materials for medical applications, in addition to supporting cell development, should also have antibacterial properties. Porous scaffolds based on polyaniline (PANI) and PHBV together with surface-immobilized curcumin were investigated for potential applications in tissue engineering. Scaffolds supported cell development, as confirmed by a very high survival rate, and showed good antimicrobial activity.^191^ Due to the numerous applications of PHA-based materials, it is worth dividing them into individual groups of tissues of living organisms as shown in Figure 13.

### 8.2 Bone Tissue Engineering

In bone tissue engineering, the porosity of the implemented material is an extremely important feature. Misra et al.^192^ created PHB foams with the addition of bioactive glass particles. Polymer foams with the addition of bioglass showed a high level of adsorbed proteins (bioactivity), while their porous microstructure supported the adhesion and proliferation of osteoblasts. The biocompatibility was also tested *in vivo*, and the produced materials, which were implanted subcutaneously in rats, showed no toxic reactions but facilitated vascularization. Moreover, it was shown that the presence of bioactive glass particles improved antibacterial properties.^192^ Composite scaffolds with a polymer matrix and the addition of bioglass can support bone reconstruction and regeneration and enhance the angiogenesis process.^193^ Wu et al.^193^ proved that the polymer composite containing 10% bioglass has great potential in the process of osteogenesis and supports vascularization compared to the other composite systems tested. Another example of the highly porous materials used for tissue engineering scaffolds is a biocomposite scaffold based on P(3HB-*co*-4HB) and bacterial cellulose.^194^ Polymer and composite scaffolds were made by freeze-drying. The participation of both phases was confirmed by tests on physicochemical properties, while mechanical properties improved after adding cellulose to the polymer. Biodegradation studies in a buffer and enzyme environment showed a much faster enzymatic than hydrolytic degradation. The differences between the degradation of PHAs and their rates are discussed in Section 4. The more hydrophilic substrate represented by the polymer–cellulose composite promoted cell adhesion and proliferation, thus making the material suitable for use in tissue engineering.^194^ Wang et al.^117^ investigated the activity and differentiation of rabbit bone marrow cells on 3D scaffolds as a potential application for bone reconstruction. *In vitro* biocompatibility was determined by comparing three types of polymer scaffolds: PHBHHx, PHB, and PLA. The greatest proliferation and deposition of calcium was observed in PHBHHx scaffolds, while the activity of alkaline phosphatase (ALP) was about two times higher than with other materials. The results confirmed the increased activity and differentiation into the osteoblasts of bone marrow cells and the possibility of using PHBHHx as a potential biomaterial for bone tissue regeneration.^117^ Ding et al.^195^ demonstrated the use of hybrid scaffolds based on polymers and inorganic particles. The electrospun fibers of the PHB/PCL blended together with the inorganic phase of 58S bioactive glass were tested to assess the bone formation properties of the scaffolds obtained. *In vitro* tests showed that the addition of the inorganic phase increased the adhesion and proliferation of osteoblasts, thus influencing osteogenic properties.^195^ An interesting example is also the bonding of HA with polymer fibers for applications in bone tissue engineering.^196^ Electrospun PHB fibers with the addition of HA were combined with a protein hydrogel to increase the porosity of the scaffold. It was found that these fibers ensured structural stability, while the use of a hydrogel provided both additional space for cell growth and an environment imitating an extracellular matrix (ECM). On the other hand, the addition of HA nanoparticles promotes mineralization in the hybrid scaffold.^196^ Rentsch et al.^111^ investigated the osteogenic properties and vascularization of PHB scaffolds. Three-dimensional scaffolds were covered with type I collagen and a mixture of collagen and chondroitin sulfate. Human mesenchymal stem cells were seeded on the scaffolds to assess cell proliferation and differentiation. *In vitro* tests showed cell proliferation in both types of scaffolds, while calcium deposition and the highest level of ALP were observed in the presence of collagen and chondroitin sulfate, which may indicate a high degree of osteogenic activity. Cell-induced scaffolds were implanted subcutaneously in rats to evaluate vascularization. Histologic examinations confirmed the presence of blood vessels and osteogenic markers. Scaffold coatings contributed to the osteogenic differentiation of human mesenchymal stem cells.^111^ The differentiation of bone marrow stromal cells was also studied using PHBHHx-based scaffolds.^112^ Fibronectin adsorption and proliferation assays confirmed cell attachment to the scaffold. The high level of ALP confirmed the ability to differentiate into osteoblasts.^197^ These preliminary results suggest a potential application of PHBHHx in bone tissue engineering.^112^

### 8.3 Cartilage Tissue Engineering

Three-dimensional PHA-based scaffolds were also tested to assess chondrocyte proliferation from articular cartilage.^101^ The porous scaffold was prepared by leaching the salt from a blend of PHBHHx and PHB as PHBHHx/PHB polymer in various ratios. Chondrocytes were isolated from the articular cartilage of the knee and hip joints of white New Zealand rabbits. The proliferation assay showed that cells grew better on PHBHHx/PHB blend scaffolds than on any homopolymer. Cells were cultured for 28 days, and in the first days of culture, the cells were seen to grow on the scaffold surface. On the other hand, from day 7, chondrocytes began to penetrate the scaffold, anchoring themselves onto the pores, while forming a cell monolayer on the surface. The different behavior of the chondrocytes can be explained by changes both in the surface morphology of the scaffolds produced and in the crystallinity of the polymer blends, thus changing the speed of the degradation process.^101^ The evaluation of PHBHHx-based scaffolds for articular cartilage repair was also investigated *in vivo*.^102^ The polymer solution was frozen and lyophilized to obtain porous scaffolds. Isolated chondrocytes from the knee and hip joints of the white Japanese rabbit were inoculated onto scaffolds for 30 days. *In vivo* studies with allogeneic chondrocytes were conducted using these scaffolds for 10 days. Histological examinations showed a filling of the cavities with white cartilage and a good connection of the subchondral bone. It was shown that the initial cultivation of chondrocytes on scaffolds promoted the accumulation of ECM and increased the integration of the surface with cartilage. Additionally, in the case of materials without precell culture, the formation of fibrous tissue around the scaffold was seen. It has been proven that PHBHHx-based scaffolds can be used in cartilage engineering, while an allogeneic cell culture supports and accelerates the reconstruction of cavities.^102^ Sun et al.^198^ investigated chondrocyte growth on macroporous PHBV scaffolds. Articular cartilage cells were isolated from the knee and hip joints of New Zealand white rabbits. *In vitro* studies found that chondrocytes retained their phenotype and exhibited a high activity for 7 days. Moreover, histological studies revealed that, with an increasing incubation time, the density of chondrocytes with their natural spherical morphology increased. In conclusion, this study showed that PHBV scaffolds can be very good carriers of chondrocytes.^198^ The cartilage tissue reconstruction phenomenon was also investigated with the use of PHBV foam matrices and calcium phosphate-containing collagen matrices (CaP-Gelfix).^103^ Both types of foams were incubated with chondrocytes isolated from the humerus of male albino rabbits. Macroscopic observations of materials, with and without cells, made it possible to determine the stability of the *in vitro* structure. The PHBV matrix was not deformed in 21 days, while the CaP-Gelfix matrix was degraded after 15 days. Scaffolds with and without chondrocytes were implanted into cartilage defects in the patella of rabbits’ knees. Histological examinations of the seeded materials showed cartilage formation and a slight inflammation reaction using the PHBV scaffold. Immediately, the use of CaP-Gelfix resulted in the formation of fibrous cartilage. Importantly, the implementation of chondrocytes on scaffolds before implantation actively influenced the formation of cartilage. It has been demonstrated that PHBV matrices can be active carriers and support the reconstruction of cartilage tissue defects.^103^

### 8.4 Nervous Tissue Engineering

**Figure 14:**
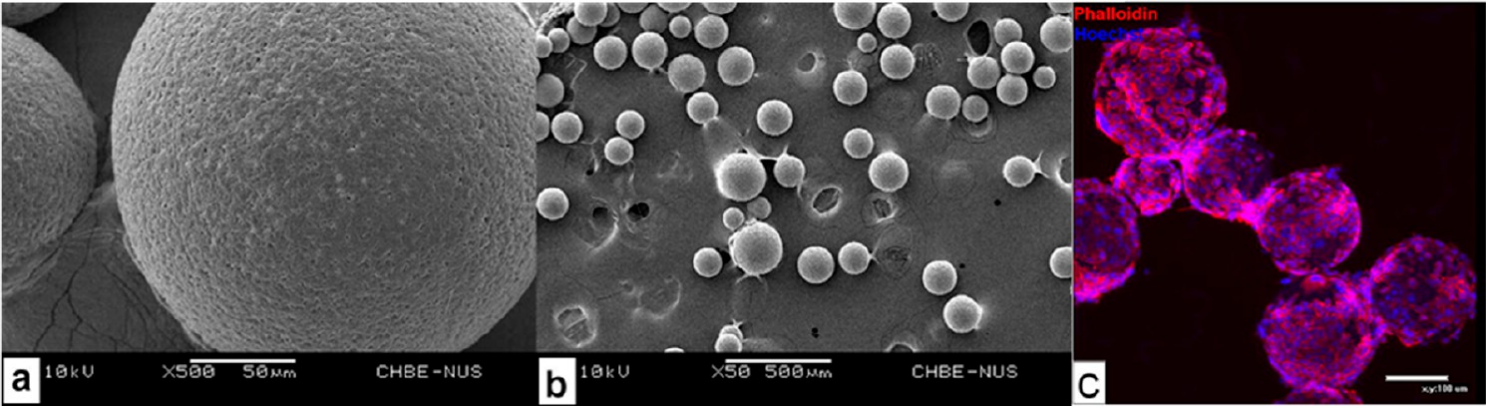
SEM images (a, b) showing the morphology of PHBV microspheres and neuronal cell growth (c) on the PHBV microspheres prepared by the solvent evaporation technique. Reproduced with permission from ref (199). Copyright 2012 Elsevier.

The use of PHA scaffolds can also include neural tissue engineering. Chen and Tong^199^ investigated the effect of PHBV microspheres on the cell growth of cortical neurons and neuronal progenitor cells. Cells grew significantly on the microspheres, compared to the coverslip control. Cell growth and proliferation were confirmed by viability tests, total DNA quantification, and immunofluorescence staining. Neuronal progenitor cells differentiated into neurons, while the expression of signal pathway markers for cortical neurons showed maturation and even created functional units (Figure 14).^199^ These results indicate that PHBV microspheres are suitable for neural tissue engineering.

**Figure 15:**
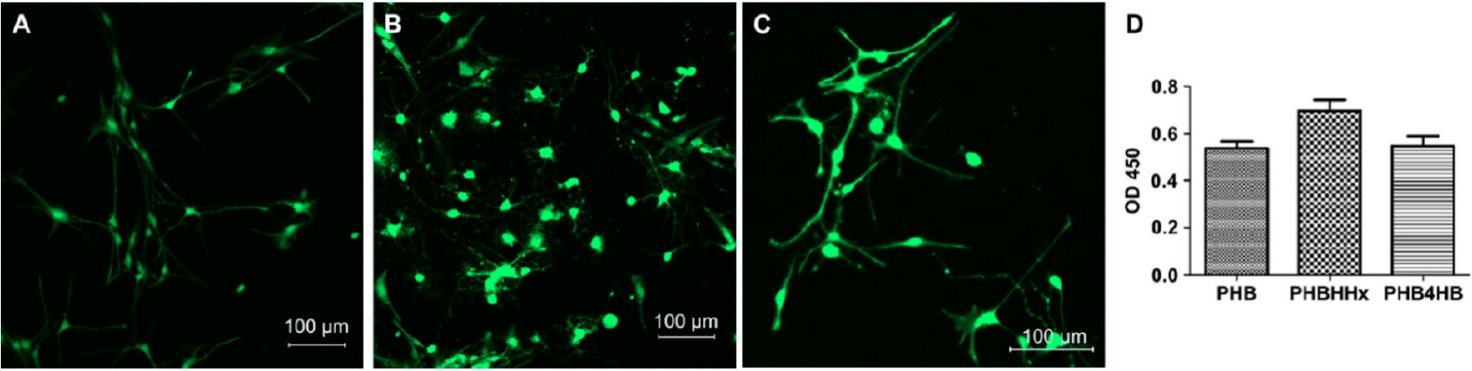
(A–C) CLSM images showing the morphology of neural stem cells on (A) PHB, (B) PHBHHx, and (C) P(3HB-*co*-4HB) fibrous scaffolds; (D) the viability assay (CCK-8) of neural stem cells on PHA scaffolds. Reproduced with permission from ref (200). Copyright 2010 Elsevier.

Spinal cord injuries lead to significant physical limitations and even disability, so repair therapies for these injuries are widely studied. It was found that PHBV, due to its properties, i.e., biocompatibility and piezoelectricity, is a suitable material supporting the regeneration of the spinal cord.^110^ 3D, porous PHBV scaffolds showed an anisotropic morphology. *In vitro* studies have confirmed their lack of cytotoxicity and the ability to promote the proliferation and growth of nerve cells from the central nervous system (CNS) and various types of mesenchymal stem cells. Conversely, the subcutaneous implantation and histological compliance examination confirmed the vascularization in the scaffold structure. Additionally, there was no rejection reaction or tissue necrosis. These results suggest that PHBV is an appropriate scaffolding material for the treatment of spinal cord injuries.^110^ Xu et al.^200^ investigated the effects of PHA fibers and flat films on neural stem cell growth and differentiation. Fibrous scaffolds have been manufactured using a phase separation method to mimic the ECM. *In vitro* studies were conducted with the use of rat neural stem cells on three materials: PHB, P(3HB-*co*-4HB), and PHBHHx (Figure 15). Cell growth and differentiation was seen on all polymeric materials: flat films and 3D scaffolds. In any case, the greatest potential for the differentiation of neural stem cells into neurons was seen on the fibrous P(HB-HHx) scaffold. Moreover, fibrous membranes, due to their 3D structure, mimicked the structure of the natural ECM and promoted cell adhesion and synaptogenesis. It has been noted that, by promoting differentiation, PHBHHx scaffolds are inexpensive materials for the treatment of central nervous system damage.^200^ Novikova et al.^201^ studied spinal cord regeneration in adult rats. Tubular scaffolds with a unidirectional fiber orientation were incubated *in vitro* with Schwann cells. The scaffolds together with the induced cells were implanted into the damaged spinal cord of adult rats. The tubular PHB scaffold was seen to be well integrated into the traumatic injury space, showing a moderate neurogliosis. Axon development was observed on the outer surface of the scaffold, while host Schwann cells did not migrate into the tube. In addition, the surface was modified with the components of the ECM, i.e., fibronectin, collagen, and laminin; nevertheless, no changes in the growth of axons were observed on the modified scaffolds. Tubular scaffolds based on PHB in combination with a preculture of Schwann cells have been shown to promote an axonal regeneration to repair spinal injuries.^201^ Another example of the use of PHA material is the model of peripheral nerve regeneration presented by Bian et al.^116^ Porous nerve conduits with homogeneous and heterogeneous wall porosity were prepared by particle leaching. The study on the permeability of bovine serum albumin, glucose, and lysozyme made it possible to conclude that the wall porosity permits the flow and exchange of nutrients. These conducts were implemented in a defect in the sciatic nerve of Sprague–Dawley rats. The increased muscle action potential, which indicated the restoration of the functionality of the damaged nerve, was observed one month after implantation. Histological studies have shown that, in the case of homogeneously porous conduits, the connective tissue overgrows the scaffold: an impossible event in the case of a dense structure of the wall surface in heterogeneous scaffolds. Biodegradability tests confirmed the nontoxicity of the degradation products, and both types of scaffolds lost about 20% of their initial molecular weight in 3 months. The PHBHHx-based conduits showed satisfactory nerve regeneration, while biodegradation properties confirm their usefulness for repairing peripheral nerve damage.^116^

### 8.5 Cardiovascular Tissue Engineering

Another interesting medical application of PHA polymers may be the production of artificial blood vessels. Cheng et al.^115^ have studied the use of rabbit blood vessel smooth muscle cells (RaSMCs) on a number of polymers, PLA, PHB, PHBHHx, and P(3HB-*co*-4HB), containing various proportions of 4HB (7–40 mol %). RaSMCs were incubated on polyester films for viability testing, while tubular scaffolds made of PHA polymers were used to assess cell morphology and density. The best viability was observed in copolymers with a 7% content of 4HB, which was confirmed by cell staining. The study of elastin cross-linked by RaSMCs was performed showing that the increased 4HB component significantly affected the formation and greater accumulation of elastin. This research showed the great potential of P(3HB-*co*-4HB) copolymers in supporting the reconstruction or development of artificial blood vessels.^115^ Qu et al.^202^ performed comprehensive studies using PHBHHx containing 0–20% HHx in contact with smooth muscle cells. *In vitro* studies were performed to evaluate the adhesion, proliferation, and phenotype changes in RaSMCs. In these studies, the best adhesion was observed in a material with a 12% HHx content, while the highest proliferation was observed in the material with a 20% HHx content. The highest level of expression and the spindle shape of α-actin were seen in a culture on a film containing 20% HHx, which indicated a tendency to differentiate RaSMCs. These research confirmed the possible potential use of PHBHHx scaffolds in contact with smooth muscle cells.^202^ In another work, Qu et al.^203^ investigated the hemocompatibility of films made of a P(3HB-3HHx) copolymer containing 4–20% 3HHx as well as with PHBV and PHB. Additionally, the surface was modified with the use of lipolase and gelatin on P(3HB-12% 3HHx) films. Surface roughness tests showed that an increase in the 3HHx content of the copolymer made the surface of PHBHHx films smoother. These materials were subjected to a hemolysis test, i.e., a test aimed at determining the breakdown of red blood cells under the influence of various factors. PHBHHx films in contact with erythrocytes showed around half the reactivity of PHB or PHBV films. Samples were incubated with platelet-rich plasma for 2 h. After 30 min of testing, no adhesion of any thrombocyte was observed on PHBHHx with 12% and 20% 3HHx and on the modified materials. After the total exposure time, only a few platelets stuck to these films, while their number on PHBV was much higher. The surface smoothness of PHBHHx with a high content of 3HHx may result in a poor platelet adhesion. The study of the metabolic activity of human umbilical vein endothelial cells (HUVECs) showed a high proliferation and biocompatibility of cells to P(3HB-12% 3HHx), P(3HB-20% 3HHx), and modified P(3HB-12% 3HHx) films. Either PHBHHx materials with a high proportion of HHx or modified materials are characterized by a high biocompatibility and low level of hemolysis, which makes them promising materials for all applications of biomaterials in contact with blood.^203^

### 8.6 Skin Tissue and Wound Healing

PHAs are also used in wound healing. Azimi et al.^97^ produced electrospun fibers from a mixture of PHB and PHOHD. In addition, in order to increase their anti-inflammatory and antimicrobial properties, meshes were electrosprayed with chitin nanofibrils and a complex of chitin nanofibrils, nanolignin, and glycyrrhetinic acid. Spectroscopic examinations confirmed the presence of the electrospray components used. *In vitro* studies on human keratinocytes confirmed the anti-inflammatory activity of meshes through their low level of proinflammatory cytokine expression.^97^ Li et al.^98^ studied PHA polymer matrices in contact with human epidermal cells: keratinocytes of the HaCaT line. PHB polymer blends with P(3HB-*co*-4HB) and PHBHHx copolymers in several ratios have been investigated. Two forms of materials were used for the research: nanofiber 3D scaffolds and matrices cast from a solution. The results of keratinocyte attachment and proliferation showed that fibrous matrices significantly support cell adhesion and proliferation. The porous structure naturally mimicked the architecture of the natural ECM of collagen. The matrices based on polymers from the PHA group can be used in skin tissue engineering.^98^

### 8.7 Liver Tissue Engineering

Tong and co-workers^104,105^ tested PHBV microspheres modified for use in liver tissue engineering. To study the activity and proliferation of human hepatoma (Hep3B) cell lines, three types of ECM proteins were attached to the PHBV microsphere.^104^ Hep3B cells were grown for 2 weeks on modified and unmodified microspheres. The attachment of ECM proteins was seen to affect the increase in cell proliferation, mimicking the environment of the liver tissue. Studies on protein combinations, however, have shown that the cellular activity does not depend on just one type of protein. These combinations influence the complex interactions between the modified material and cells. Moreover, it has been observed that, with a low hepatocyte proliferation, cells are rounder but their liver functions performed better. However, a high proliferation limited the functionality of cells.^104^ Another example of the use of polymer microspheres in liver tissue engineering is the encapsulation of bovine serum albumin (BSA) and hepatocyte growth factor (HGF).^105^ This study determined the ability to provide growth factors supporting tissue regeneration by using microspheres as a cell scaffold. These microspheres were made of PHBV, PLGA, and a blend of both polymers. Due to its molecular weight being similar to that of HGF, BSA acted as a model protein. In addition, BSA prevented growth factor denaturation. Composite microspheres (PHBV/PLGA) degraded slower than PLGA and faster than PHBV ones alone. The BSA release rate from composite microspheres was also averaged and compared to the homopolymers. In summary, microspheres with encapsulated growth factor improve the phenotype of hepatocytes.^105^

### 8.8 Drug Delivery Systems

The variety of processes used for polymeric materials has resulted in an increase in their potential applications. In addition to films, foams, or fibers, as described above, it is also possible to create polymer microspheres. Wang et al.^204^ developed a drug release system based on composite microspheres of PHBV and HA. HA particles and the drug were encapsulated in polymer casings. At the beginning, a low level of release was recorded, but later, the drug diffusion phase was higher and stable. It has been found that microspheres for long-term drug delivery and release can be produced by using PHBV and HA, due to the stable and long-term degradation of PHBV compared to other polymers.^204^ The controlled release of the drug from PHBV microspheres was also investigated by Rossi et al.^205^ A continuous antibiotic delivery system has been developed to treat local orthopedic infections. Gentamicin is a bactericidal antibiotic; it has been enclosed in PHBV microspheres with 8% or 12% HV content. It has been shown that a faster drug release occurs in microspheres with a higher content of 3HV, which may be related to the degree of polymer crystallinity (see Figure 4). Bactericidal properties were tested in contact with *Staphylococcus hemolyticus* and *Staphylococcus aureus*. The released gentamicin caused a significant decrease in bacterial adhesion in contact with microspheres. It can be concluded that microspheres prevent bacterial infections and may have potential applications in the treatment of orthopedic infections.^205^ The release of gentamicin from PHB polymer microspheres was also investigated by Francis et al.,^206^ who encapsulated gentamicin into microspheres produced by the solid-in-oil-in-water method. The surface morphology and properties were examined as well as the size of the microspheres, depending on the production parameters. The kinetics of gentamicin release from microspheres into simulated body fluid (SBF) was investigated. Thanks to the hydrophilic nature of gentamicin, the drug release was rapid in the initial stage, whereas later, diffusion became stable. These results demonstrate the potential use of PHB-based microspheres as drug carriers, especially for the release of potent drugs used in therapy.^206^ PHB microspheres modified with gelatin and a mixture of methoxy PEG and P(d,l-LA) were developed as a controlled ibuprofen release system.^207^ The modifications in microspheres were intended to slow down and prolong the drug release process. Composite microspheres with the addition of gelatin did not affect the possibility of a controlled release. Microspheres of PHB and a mixture of methoxy PEG and P(d,l-LA) were produced in two ratios, 1:1 and 3:1. In the 1:1 blend, a reduced spread in the drug release was observed, while with the 3:1 blend, the ibuprofen release system was more time-controlled.^207^ Peng et al.^208^ designed an insulin release system based on biodegradable PHBHHx nanoparticles. The insulin–phospholipid complex was encapsulated into PHBHHx by solvent emulsion evaporation. *In vitro* drug release studies showed that only 20% of the drug was released from the nanoparticles with more than 5% in the first 8 h. Conversely, *in vivo* studies in diabetic rats showed that the reduction in blood sugar after the subcutaneous injection of nanoparticles persisted for more than 3 days. The sustained drug release system from the nanoparticles is a promising result. This is a significant difference from the oral administration of insulin; with the subcutaneous injection system of the tested complex, the frequency of drug administration can be significantly reduced. Studies of PHBHHx-based nanoparticles with an insulin–phospholipid complex confirmed that it is a suitable material for the controlled delivery of insulin or other hydrophilic drugs.^208^ Information on the studied blends of PHA polymers with other polymers and particulates for medical applications is summarized in Table 3.

**Table 3:** Summary of PHAs and Their Blends in Medical Applications

application or properties	polymer blends and particles	main type of studies	reference
gastrointestinal patches	PHB/PLLA blends	*in vitro* and *in vivo* degradations	(114)
surface for cell culture tests	PHB and PHBV scaffolds	*in vitro*: fibroblasts (NIH 3T3)	(185)
	P(3HB-*co*-4HB) blend with cellulose	*in vitro*: Chinese Hamster Lung (CHL) cells	(194)
bone scaffolds	PHB with bioglass particles	*in vitro*: osteoblasts (MG-63); *in vivo*: rat model	(192)
	PHBHHx, PHB, and PLA	*in vitro*: bone marrow cells isolated from rabbits	(117)
	PHB/PCL blend with bioglass particles	*in vitro*: osteoblasts (MG-63)	(195)
	PHB with HA	*in vitro*: preosteoblasts (MC3T3-E1)	(196)
	PHB with collagen	*in vitro*: human mesenchymal stem cells (hMSCs); *in vivo*: rat model	(111)
	PHBHHx scaffolds	*in vitro*: human bone marrow cells	(112)
cartilage engineering	PHBHHx-PHB blends	*in vitro*: chondrocytes isolated from rabbit articular cartilages	(101)
	PHBV scaffolds	*in vitro*: chondrocytes isolated from rabbit articular cartilages	(198)
neural tissue engineering	PHBV microspheres	*in vitro*: PC12 cells, cortical neurons (CNs), and neural progenitor cells (NPCs)	(199)
	PHBV scaffolds	*in vitro*: mix primary culture of neurons and astrocytes from the hippocampus of Wistar rats	(110)
	PHB, P(3HB-*co*-4HB), and PHBHHx scaffolds	*in vitro*: neural stem cells (NSCs)	(200, 209)
artificial blood vessels	PLA, P(3HB), PHBHHx, and P(3HB-*co*-4HB) blends	*in vitro*: rabbit blood vessel smooth muscle cells (RaSMCs)	(115)
	PHBHHx blends	*in vitro*: smooth muscle cells from rabbit aorta (RaSMCs)	(202)
wound healing	P(3HB)/PHOHD blends	*in vitro*: keratinocytes (HaCaT)	(97)
	PHB/P(3HB-*co*-4HB)/PHBHHx blends	*in vitro*: keratinocytes (HaCaT)	(98)
liver tissue engineering	PHBV microspheres	*in vitro*: human hepatoma cell line (Hep3B)	(104, 105)
antibacterial properties	PAni-PHBV blend with curcumin	hemolytic assays, antimicrobial activity, *in vitro*: fibroblasts (NIH 3T3)	(191)
	PHBV microspheres with gentamicin	hemolytic assays, antimicrobial activity, drug release test	(205)
	P(3HB) microspheres with gentamicin	BSA adsorption test, drug release test	(206)
drug release system	P(3HB) microspheres with ibuprofen	drug release test	(207)
	PHBHHx particles with insulin	drug release test, *in vivo*: rat model of diabetes	(208)

## 9 Conclusions and Future Prospects

PHA polymers show great potential for medical applications, as their biodegradability and biocompatibility make them ideal candidates for the treatment and regeneration of tissues. Importantly, these polymers support cell proliferation as well as their growth on scaffolds, which makes them suitable for use in medical devices and tissue engineering. Scaffolds, in their various forms, are tested for the regeneration of bone and cartilage tissue, while films and fibers are more often used in wound healing, and polymer microspheres are used in drug delivery and nerve repair systems. Within this Review, we showed that creating blends from various PHA polymers or incorporating particles into the polymer structure by using electrospinning really expands the applications of these materials. The scaffolds developed with other synthetic and natural polymers and the use of particles of other phases can improve the mechanical and biological properties of the PHA scaffolds. Electrospun polymer fibers have an advantage over film, because of their larger surface area, which is conducive to either the incorporation of particles of a different phase or an increased adsorption and immobilization of other active substances. Numerous innovations in the production of electrospun fibers have been introduced in recent years. However, many challenges still remain with regard to the electrospinning of more complex materials in a reproducible manner.

Depending on requirements, polymers from the PHA group can be mixed, surface-modified, and combined with other polymers or inorganic materials to improve and extend their properties. Mixing just two PHA polymers can change the material’s properties and potential use. An example of this is the PHBV polymer which, in its basic form, is a combination of two monomers: HB and HV. This combination, by changing the structure of the polymer, was proven to affect the crystallinity, thermal and mechanical properties and, importantly, the rate of material degradation. In addition, the presented examples of PHBV modifications with other synthetic and natural polymers have increased the spectrum of applications, in particular by increasing the biocompatibility of the material, improving the cellular response, and reducing the inflammatory response *in vivo*. Nevertheless, both mechanical and biological properties can be improved by surface or volumetric modification. The electrospinning process offers many possibilities for changes in the one-stage process of producing a material with the desired properties in the biomedical field.

There is a great need to slow down the production of waste and replace petroleum-based plastics with biodegradable materials of natural origin. PHAs are interesting polymers that can be used extensively in industrial fields. However, there is an efficiency problem, as the bacterial production of polymers is constantly being developed. Another disadvantage that scientists all over the world are working on is the improvement of the mechanical and thermal stability properties of PHAs. In summary, this Review shows the continuous need of development and improvement of PHA-based materials based on the PHBV modifications and manufacturing scaffolds and membranes via electrospinning.

## Abbreviations

PHBV, P(3HB-*co*-3HV): poly(3-hydroxybutyrate-*co*-3-hydroxyvalerate)
PHA: polyhydroxyalkanoate
PLA: polylactide
PLLA: poly(l-lactide)
P(d,l-LA): poly(d,l-lactide)
PGA: polyglycolide
PCL: polycaprolactone
PHB: polyhydroxybutyrate
P(3HB): poly(3-hydroxybutyrate)
PLGA: poly(lactic-*co*-glycolic acid)
HB: hydroxybutyrate
HV: hydroxyvalerate
HHx: hydroxyhexanoate
SCL: short-chain length
MCL: medium-chain length
P(4HB): poly(4-hydroxybutyrate)
P(3HO): poly(3-hydroxyoctanoate)
P(3HHx-*co*-3HO): poly(3-hydroxyhexanoate-*co*-3-hydroxyoctanoate)
LDPE: polyethylene
PS: polystyrene
PP: polypropylene
P(3HB-*co*-4HB): poly(3-hydroxybutyrate-*co*-4-hydroxybutyrate)
PBS: phosphate buffered saline
DMF: dimethylformamide
TFE: 2,2,2-trifluoroethanol
HFIP: 1,1,1,3,3,3-hexafluoro-2-isopropanol
DCM: dichloromethane
CFO: cobalt ferrite
TFA: trifluoracetic acid
PEO: poly(ethylene oxide)
HA: hydroxyapatite
PGS: poly(glycerol sebacate)
PHBHHx, P(HB-*co*-HHx): poly(hydroxybutyrate-*co*-hydroxyhexanoate)
PEG: poly(ethylene glycol)
PET: poly(ethylene terephthalate)
PAni: polyaniline
ALP: alkaline phosphatase
ECM: extracellular matrix
CNS: central nervous system
RaSMC: rabbit blood vessel smooth muscle cells
PHOHD: poly(3-hydroxyoctanoate-*co*-3-hydroxydecanoate)
BSA: bovine serum albumin
HGF: hepatocyte growth factor
SBF: simulated body fluid
EDX: energy dispersive X-ray analysis
PLL: poly-l-lysine
hFob: human fetal osteoblast
CrO_2_: cerium oxide
HOEC: human oral epithelial cells
HMEC: human mammary epithelial cells
ZnO: zinc oxide
CNC: cellulose nanocrystal
MSCs: mesenchymal stem cells
